# NMR spectroscopic evidence that the antileishmanial drug sodium stibogluconate comprises one predominant molecular species^☆^

**DOI:** 10.1016/j.jinorgbio.2026.113237

**Published:** 2026-01-17

**Authors:** Alissa Lance-Byrne, Juliet C. Gee, Timothy C. Johnstone

**Affiliations:** a Molecular, Cellular, and Developmental Biology, University of California Santa Cruz, Santa Cruz, California 95064, United States; b Department of Chemistry and Biochemistry, University of California Santa Cruz, Santa Cruz, California 95064, United States

**Keywords:** Leishmaniasis, Antimony, Pentavalent antimonial, Stibogluconate, Pentostam^®^, Hexahydroxoantimonate

## Abstract

Sodium stibogluconate is an effective but toxic Sb-containing antileishmanial drug. Despite having been in clinical use for over half a century, the chemical structure of this small-molecule drug remains unknown. Historically, the drug has been thought to comprise an intractable mixture of interconverting species. We report here nuclear magnetic resonance (NMR) spectroscopic experiments that provide the first evidence that the reaction between gluconate and [Sb(OH)_6_]^−^ produces primarily one molecular species. Multidimensional experiments allow the NMR resonances of this species to be fully assigned. Further experiments on authentic samples and clinical preparations of sodium stibogluconate confirm that the primary product of the reaction of gluconate and [Sb(OH)_6_]^−^ is the predominant antimony-containing component of the drug. The thermodynamic stability of this predominant species was assessed using a combination of ^1^H and ^121^Sb NMR spectroscopic measurements, which afforded a value of *K* = 1006 M^−1^ for its formation from gluconate and [Sb(OH)_6_]^−^.

## 1. Introduction

**Fig. 1.**
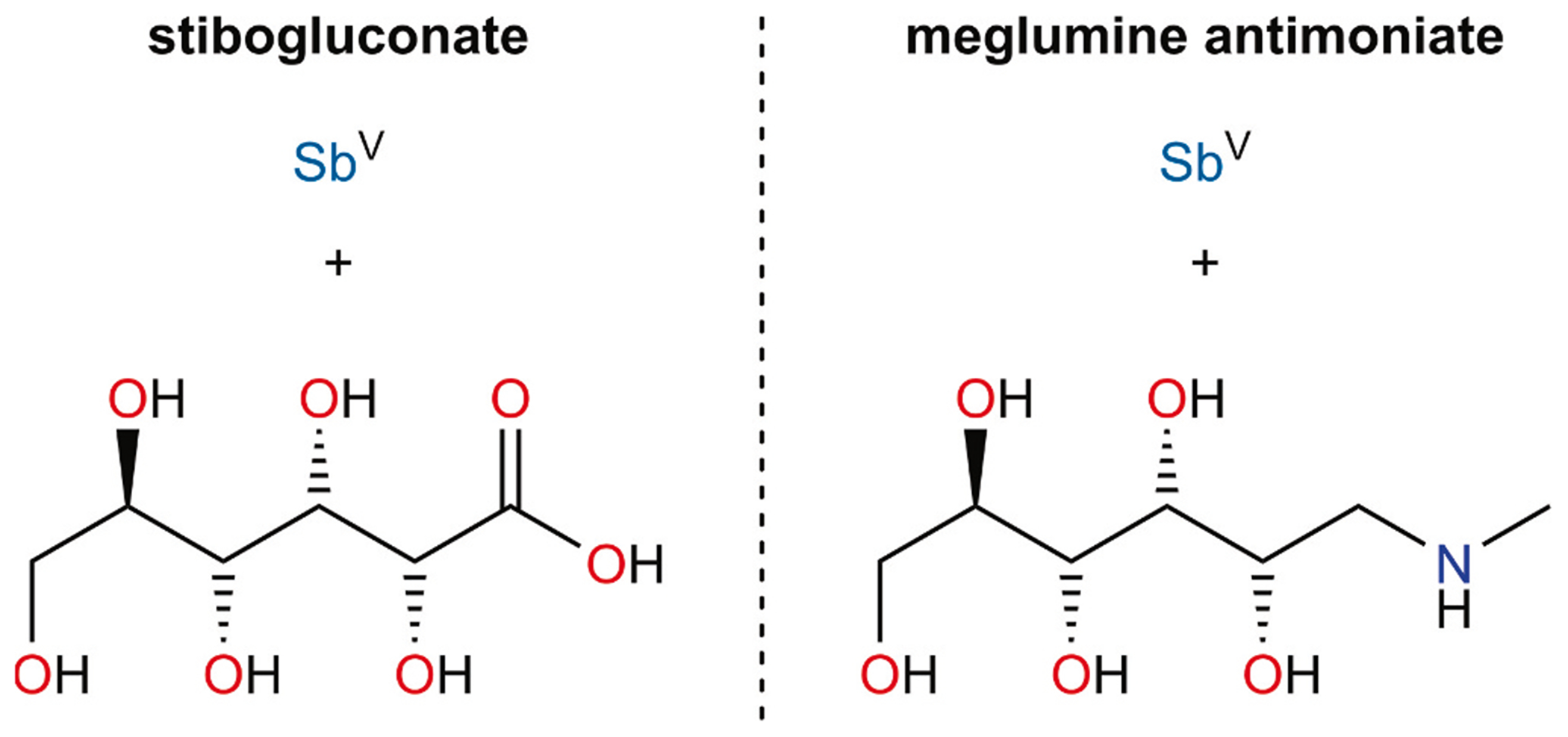
The components of the clinically used pentavalent antimonial drugs.

Leishmaniasis is a neglected tropical disease caused by infection with protozoal parasites from the genus Leishmania. Over 200,000 new cases of *leishmaniasis* requiring individual case management occur each year [1,2], but the World Health Organization estimates that up to 1,000,000 new cases occur annually [3]. As many as 90,000 of these estimated new cases comprise the visceral form of the disease, which is lethal in >95% of cases if left untreated [3]. Currently, two Sb(V) compounds, known collectively as the *pentavalent antimonials*, are marketed for frontline use in the treatment of leishmaniasis: sodium stibogluconate and meglumine antimoniate [4]. Each of the drugs features Sb(V) chelated by a carrier ligand. The carrier ligand in sodium stibogluconate is d-gluconate (hereafter simply referred to as gluconate), and that in meglumine antimoniate is *N*-methyl-d-glucamine (Fig. 1). These drugs have potent parasiticidal activity and are typically highly effective in treating leishmanial infections, but they are also plagued by high toxicity to the patient [5]. Important work is being done to discover new classes of antileishmanial compounds [6,7], but the fact remains that the cost-effective Sb-based drugs are the present-day standard frontline therapy in many of the low- and middle-income countries impacted by the disease [8,9]. Antimonial drugs will continue to be clinically employed for the foreseeable future. As such, there remains a pressing need to gain a greater understanding of their chemistry and biology. A key step in gaining that understanding will be ascertaining the molecular compositions of the pharmaceutically active ingredients in these drugs. Progress has been made in understanding the fundamental biochemistry of these drugs [10], but further development has been significantly hampered by the fact that, despite having been in clinical use for decades, their composition remains unknown [4]. The pentavalent antimonials, which are prepared by combining a source of Sb(V) with the appropriate chelating ligand [11], are commonly described as intractably complex mixtures of interconverting species with unknown structures [12]. This conclusion has been informed by mass spectrometric experiments that confirm the interaction of the carrier ligand with Sb, but feature a large number of signals whose *m*/*z* values indicate variable numbers of ligands and Sb atoms [11,13]. The labile nature of the Sb–OR bond could, however, result in rearrangement during ionization, and such data should be interpreted with caution [14].

Here we report a detailed NMR spectroscopic investigation of products of the reaction of K[Sb(OH)_6_] with potassium gluconate, a salt of the carrier ligand used in the preparation of stibogluconate. The resulting spectra are complex and show that more than one product is generated but, contrary to the prevailing paradigm, the mixture was found to comprise primarily one molecular species. A full assignment of the ^1^H and ^13^C NMR signals for this species, as well as for one of the minor products, was carried out. Further spectroscopic experiments performed on authentic samples of sodium stibogluconate and on clinical preparations of the drug (Pentostam^®^) confirm that the predominant Sb-containing species in each is the same as the primary product from the reaction of K[Sb(OH)_6_] with potassium gluconate.

## 2. Experimental

### 2.1. General methods

Reagents and solvents were purchased from commercial vendors and used as received unless otherwise specified. (PPh_4_)[SbCl_6_] was prepared as described previously [15]. Na[Sb(OH)_6_] was prepared by combining aqueous solutions of K[Sb(OH)_6_] and NaCl in a manner analogous to one described previously [16]. The reaction of K[Sb(OH)_6_] and potassium gluconate is derived from previously reported procedures for the synthesis of meglumine antimoniate and sodium stibogluconate [11,17]. In all reactions of K[Sb(OH)_6_] with potassium gluconate, the pD of the final reaction mixture was confirmed to be 7 using colorimetric indicator paper. NMR spectra were collected using either a Bruker Avance III HD 500 spectrometer equipped with a multinuclear Smart Probe or a Bruker Avance III HD 800 MHz spectrometer equipped with a TCI cryoprobe. Signals in the ^1^H and ^13^C{^1^H} NMR spectra are reported in ppm as chemical shifts from tetramethylsilane and were referenced using dimethylsulfone (^1^H, 3.14 ppm; ^13^C, 42.2 ppm) or acetone (^1^H, 2.22 ppm; ^13^C, 215.94 ppm, 30.89 ppm) as an internal standard. The frequencies of ^121^Sb{^1^H} NMR signals are reported in ppm as chemical shifts from (PPh_4_)[SbCl_6_] (referenced to a 66.8 mM DMF solution of (PPh_4_)[SbCl_6_] in a coaxial insert at 0 ppm). Mass spectrometric experiments were performed on an Agilent model 6130 single-quadrupole instrument.

### 2.2. Mass spectrometric investigation of the reaction of potassium gluconate with K[Sb(OH)_6_]

K[Sb(OH)_6_] (13 mg, 0.05 mmol) was added to H_2_O (750 μL) and stirred at 70 °C until fully dissolved. Potassium gluconate (12 mg, 0.05 mmol) was added to H_2_O (250 μL) and stirred until fully dissolved. These two solutions were combined, and the reaction mixture was stirred for 2 h at 70 °C. The homogeneous solution was cooled to room temperature, diluted 200-fold, and directly injected into an Agilent 6130 single-quadrupole mass spectrometer.

### 2.3. NMR spectroscopic investigation of the reaction of potassium gluconate with increasing amounts of K[Sb(OH)_6_]

Three equivalent solutions of potassium gluconate (7 mg, 0.028 mmol, dissolved in 1 mL D_2_O) were prepared. Three solutions of K[Sb(OH)_6_] were prepared by adding 15 mg (0.057 mmol, 2 equiv), 30 mg (0.114 mmol, 4 equiv), or 60 mg (0.228 mmol, 8 equiv) to D_2_O (4 mL) and stirring at 70 °C until fully dissolved. Once dissolved, the K[Sb(OH)_6_] solutions were cooled to room temperature, combined with the gluconate solutions, and allowed to stand at room temperature for at least 27 h before ^1^H NMR spectra were collected.

### 2.4. NMR spectroscopic investigation of the reaction of K[Sb(OH)_6_] with excess potassium gluconate

K[Sb(OH)_6_] (13 mg, 0.05 mmol) was added to D_2_O (750 μL) and stirred at 70 °C until fully dissolved. Potassium gluconate (118 mg, 0.5 mmol) was added to D_2_O (250 μL) and stirred until fully dissolved. These solutions were combined, stirred for 2 h at 70 °C, cooled to room temperature, and then a ^13^C{^1^H} NMR spectrum was collected.

### 2.5. Spectroscopic characterization of the product of the reaction of potassium gluconate and K[Sb(OH)_6_]

Potassium gluconate (7 mg, 0.028 mmol) was added to D_2_O (1 mL) and stirred until fully dissolved. K[Sb(OH)_6_] (60 mg, 0.228 mmol) was added to D_2_O (4 mL) and stirred until fully dissolved. These solutions were combined, stirred for 2 h at 70 °C, cooled to room temperature, and then ^1^H, ^13^C{^1^H}, ^1^H–^1^H correlation spectroscopy (COSY), ^1^H–^13^C heteronuclear single quantum coherence (HSQC), and ^1^H–^13^C heteronuclear multiple bond correlation (HMBC) NMR spectra were collected.

### 2.6. Spectroscopic characterization of the product of the reaction of potassium gluconate and Na[Sb(OH)_6_]

Potassium gluconate (70 mg, 3 mmol) was added to D_2_O (1 mL) and stirred until fully dissolved. Na[Sb(OH)_6_] (12 mg, 0.05 mmol) was added to the solution of potassium gluconate. Material fully dissolved after 30 min. The reaction was allowed to stir for an additional 2 h at 70 °C, cooled to room temperature, and then ^1^H and ^13^C{^1^H} NMR spectra were collected.

### 2.7. Spectroscopic characterization of authentic sodium stibogluconate

Commercial sodium stibogluconate (10 mg) was dissolved in D_2_O (1 mL) and stirred at 70 °C until fully dissolved. The solution was cooled to room temperature and a ^1^H NMR spectrum was collected. K[Sb(OH)_6_] (13 mg, 0.05 mmol) was added to D_2_O (1 mL) and stirred at 70 °C until fully dissolved. This solution was added to the commercial stibogluconate solution, and the combined solution was stirred for 2 h at 70 °C. The solution was cooled to room temperature and then ^1^H and ^13^C{^1^H} NMR spectra were collected.

### 2.8. Simulation of ^1^H NMR spectra

^1^H NMR spectral simulation was performed using the spin simulation module of the Mnova NMR software package.

### 2.9. Calibration curve for quantification of [Sb(OH)_6_]^−^ by ^121^Sb NMR

K[Sb(OH)_6_] solutions were prepared by dissolving 18 mg (0.068 mmol), 15 mg (0.057 mmol), 12 mg (0.046 mmol), 9 mg (0.034 mmol), 6 mg (0.023 mmol), 3 mg (0.011 mmol), or 1 mg (0.004 mmol) in D_2_O (700 μL). For each sample, a ^121^Sb{^1^H} NMR spectrum was collected using (PPh_4_)[SbCl_6_] (66.8 mM) as an integration standard (Fig. S14). For all samples, the pulse width was 10 μs, the relaxation delay was 150 ms, 256 scans were averaged, the receiver gain was 203, and 17,855 data points were acquired. Integrations of K[Sb(OH)_6_] relative to (PPh_4_) [SbCl_6_] afforded a linear calibration curve (Fig. S15).

### 2.10. Quantification of the equilibrium interaction between gluconate and [Sb(OH)_6_]^−^

Equimolar solutions of K[Sb(OH)_6_] and potassium gluconate in D_2_O were prepared as described above. These solutions were combined in the appropriate proportions to give solutions with initial (identical) K[Sb(OH)_6_] and potassium gluconate concentrations of 55, 45, 35, 25, and 15 mM. The solutions were heated to 70 °C for 2 h and then allowed to stand at room temperature for 14 d before ^1^H and ^121^Sb{^1^H} NMR spectra were collected (Figs. S16, S17). Free gluconate was quantified by integrating the H2 signal at 4.12 ppm and the primary Sb–gluconate product was quantified by integrating the H5 signal at 4.07 ppm; in both cases, the concentration in solution was obtained by comparison to the integral of a dimethylsulfone internal standard (46.45 mM) signal at 3.14 ppm. The [Sb(OH)_6_]^−^ concentration was obtained by comparing the integral of the signal at 296 ppm to that of a coaxially inserted solution of (PPh_4_)[SbCl_6_] as described above with the exception that the number of scans was increased to 4096.

### 2.11. X-ray crystallography

Crystals of K[Sb(OH)_6_] were obtained by vapor diffusion of acetone into an aqueous solution of the compound. Crystals of (PPh_4_)[SbCl_6_] were obtained by vapor diffusion of diethyl ether into a DMF solution of the compound. In each case, a diffraction-quality crystal was selected under a microscope, loaded onto a MiTeGen polyimide sample loop using type NVH Cargille immersion oil, and mounted onto a Rigaku XtaLAB Synergy-S single-crystal diffractometer. Each crystal was cooled to 100 K under a stream of nitrogen. Diffraction of Cu Kα radiation from a PhotonJet-S microfocus source was detected using a HyPix6000HE hybrid photon counting detector. Screening, indexing, data collection, and data processing were performed with CrysAlis^Pro^ [18]. The structures were solved using SHELXT and refined using SHELXL following established strategies [19–21]. All non-H atoms were refined anisotropically. In the structure of K[Sb(OH)_6_], the O-bound H atoms were placed at calculated positions and refined using a riding model in which the torsion about the Sb–O bond was allowed to refine. The Uiso of each H atom was set equal to 1.5 × U_eq_ of the O atom to which it was bound. In the structure of (PPh_4_)[SbCl_6_], H atoms were placed at calculated positions, and refined using a riding model with U_iso_ equal to 1.2 × U_eq_ of the C atom to which it was bound. The structure of K[Sb(OH)_6_] has metric symmetry that suggests a crystal system that is higher symmetry than monoclinic, but the Laue symmetry was confirmed to be 2/*m* and not *mmm*. The structure was, however, twinned (reflection across the *bc* plane; final refined BASF: 0.2343(10)).

## 3. Results and discussion

### 3.1. Mass spectrometric investigation of the reaction of potassium gluconate with K[Sb(OH)_6_]

The pentavalent antimonial drugs are prepared by combining an Sb (V) precursor with an appropriate carrier ligand [11]. For sodium stibogluconate, the carrier ligand is gluconate and [Sb(OH)_6_]^−^ is the Sb(V) source. As implied in its name, sodium stibogluconate would be formed by combining the sodium salts of these anions [17]. The high hydration enthalpy of Na^+^ cations generally results in most sodium salts being highly water soluble, but Na[Sb(OH)_6_] is characteristic in that it is one of the few sodium salts that has relatively low solubility in water (3.3 mM at 298 K) [22]. To ensure that a wide range of concentrations and reactant ratios could be studied, we instead investigated the reaction of potassium gluconate with K[Sb(OH)_6_]. Data presented in Section 3.4 confirm that the same Sb-containing products form regardless of whether Na^+^ or K^+^ is the counterion.

Although K[Sb(OH)_6_] dissolves slowly at room temperature, heating to 70 °C allowed solutions with concentrations up to 75 mM to be prepared within 20 min. The solutions were stable, and no solid precipitated upon cooling to room temperature. A 1:1 solution of K[Sb(OH)_6_] and potassium gluconate was heated at 70 °C for 2 h and analyzed by electrospray ionization mass spectrometry (ESI-MS) (Fig. S1). The negative ion mode spectrum agreed well with that previously reported [17]. Sb has two abundant stable isotopes (57% ^121^Sb and 43% ^123^Sb), which gives any Sb-containing species a characteristic isotopic distribution pattern in the mass spectrum. As noted by previous investigators [17], there are a number of Sb-containing signals in the spectrum, the *m*/*z* values of which imply not only the presence of a multitude of species but that some among these species feature high degrees of coordinative unsaturation and/or oligomerization (Fig. S1). These mass spectrometric data are invaluable insofar as they confirm that the carrier ligand is bound to Sb, but care should be taken in concluding that the gas-phase ions detected in this experiment correspond directly to the species present in solution. Values of *m*/*z* that suggest coordinative unsaturation are common with coordination compounds whose dative interactions can break during the ionization process, even with gentle techniques like ESI. Potentially more concerning is that the apparent oligomers could arise from recombination following fragmentation during the ionization process [23].

To assess the likelihood of such factors arising, we acquired an ESI mass spectrum of a solution of K[Sb(OH)_6_] under conditions identical to those described above (Fig. S2). The speciation of [Sb(OH)_6_]^−^ as a function of potential and pH has been studied, and under aerobic conditions it exists almost exclusively as the hexacoordinate [Sb(OH)_6_]^−^, except at very low pH values [24]. The mass spectrum of the K[Sb(OH)_6_] solution shows an almost negligible signal for [Sb(OH)_6_]^−^, a number of signals for increasingly dehydrated species, and a cluster of signals for oligomerized species (Fig. S2). To more deeply interrogate these compounds as they exist in solution, we therefore turned to NMR spectroscopic experiments.

### 3.2. NMR spectroscopic investigation of the reaction of potassium gluconate with increasing amounts of K[Sb(OH)_6_]

**Fig. 2.**
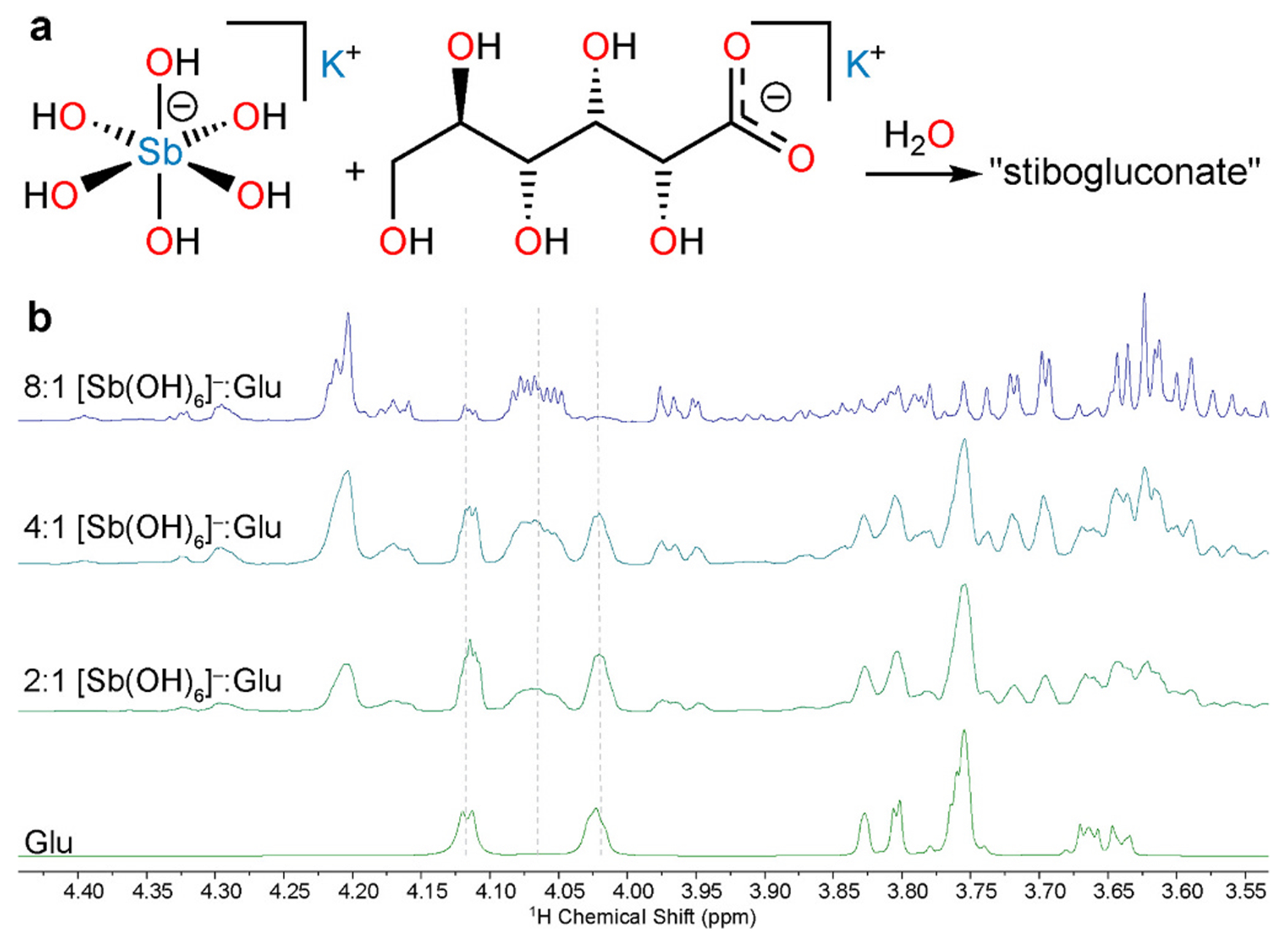
a) Reaction reported to generate stibogluconate, the composition and structure of which are still unknown. b) ^1^H NMR spectra (500 MHz, D_2_O, pD 7) for potassium gluconate (Glu) and mixtures of K[Sb(OH)_6_] and potassium gluconate in the indicated ratios.

A combination of equimolar D_2_O solutions of K[Sb(OH)_6_] and potassium gluconate (final concentration 50 mM of each, pD 7) at room temperature afforded a colorless homogeneous mixture. An NMR spectrum of the reaction mixture acquired immediately after it was prepared featured only signals from unreacted gluconate (Fig. S4). If the mixture was allowed to stand at room temperature, new ^1^H NMR signals appeared over the course of one day, although unreacted gluconate remained. If heated to 70 °C for 2 h and then cooled to room temperature, the same sets of signals were present in the ^1^H NMR spectrum (Fig. S5). Increasing the reaction time beyond 2 h did not result in any further progress in the reaction (Figs. S6, S7). If the reaction was repeated with the same amount of potassium gluconate but systematically increasing initial amounts of K[Sb(OH)_6_] (11.4–45.6 mM), there was a systematic decrease in the intensity of the signals of unreacted gluconate and a further growth of the new signals (Fig. 2). The free gluconate signals were effectively reduced to baseline intensity at an 8:1 ratio of K[Sb(OH)_6_] to potassium gluconate.

As with the ^1^H NMR data, the ^13^C{^1^H} NMR spectrum for the 1:1 reaction of gluconate and [Sb(OH)_6_]^−^ showed signals from residual unreacted gluconate (Figs. S8, S9). At the higher levels of [Sb(OH)_6_]^−^, the free gluconate signals were eliminated, and one primary set of six product signals remained. One other set of six signals was present, albeit at a much lesser intensity, and a further two sets of signals were present at such low intensity that they could only be observed upon averaging >12,000 scans. The ^13^C{^1^H} NMR data clearly indicated that the reaction between K[Sb(OH)_6_] and potassium gluconate produced one primary product, which featured a single gluconate environment.

### 3.3. NMR spectroscopic investigation of the reaction of potassium gluconate with increasing amounts of K[Sb(OH)_6_]

**Fig. 3.**
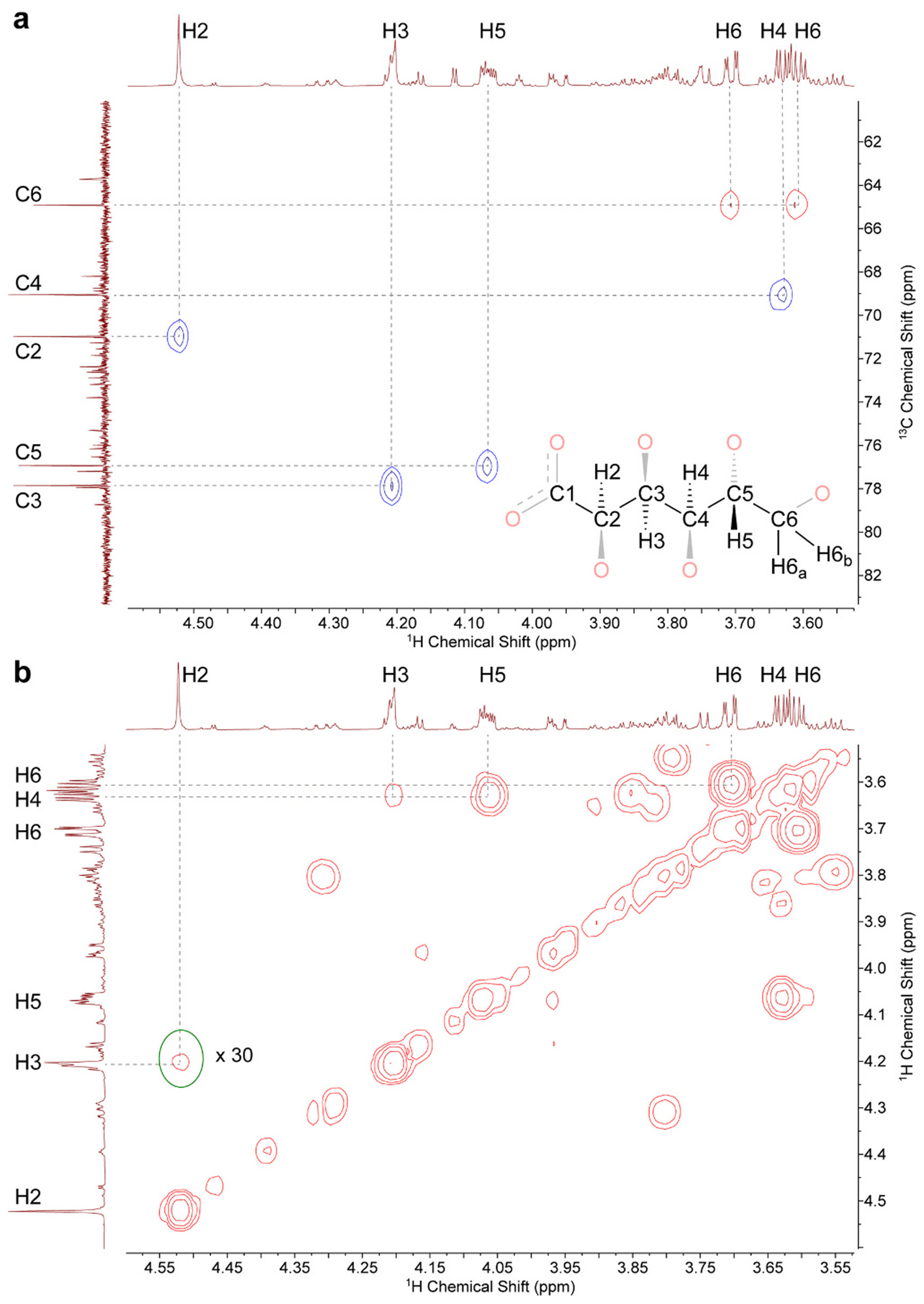
a) ^1^H–^13^C HSQC and (b) ^1^H–^1^H COSY NMR spectra (800 MHz, D_2_O, pD 7) of an 8:1 mixture of K[Sb(OH)_6_] and potassium gluconate. The circled region in (b) has been magnified 30-fold.

As the amount of [Sb(OH)_6_]^−^ was increased, there was no difference in the number or distribution of products, but rather only a systematic increase in the conversion of the unreacted gluconate. As noted above, at an 8:1 ratio of [Sb(OH)_6_]^−^ to potassium gluconate, the signals from unreacted gluconate were effectively removed from the ^1^H NMR spectrum, simplifying it significantly. A ^1^H–^13^C HSQC experiment on this mixture readily revealed which of the ^1^H signals correspond to the primary product (Fig. 3a). The only negatively phased HSQC signals arise from the sole CH_2_ group, H6_a_ and H6_b_. In a ^1^H–^1^H COSY experiment (Fig. 3b), these signals expectedly coupled most strongly with each other and with only one other signal, which identified it as H5. The only other signal to which H5 coupled in the COSY spectrum was identified as H4, and it is notable from the relative magnitudes of the cross-peaks that ^3^*J*_H4-H5_ is much greater than either ^3^*J*_H5-H6a_ or ^3^*J*_H5-H6_b. The H4 signal has only one other cross-peak, which identified H3. The one remaining ^1^H signal from the primary product (that of H2) is a singlet and has only an extremely weak COSY cross-peak with H3. With the assignments of all the H atoms complete, the remaining C signals in the HSQC could be assigned. These assignments were consistent with ^1^H–^13^C HMBC data that were additionally collected (Fig. S10).

**Fig. 4.**
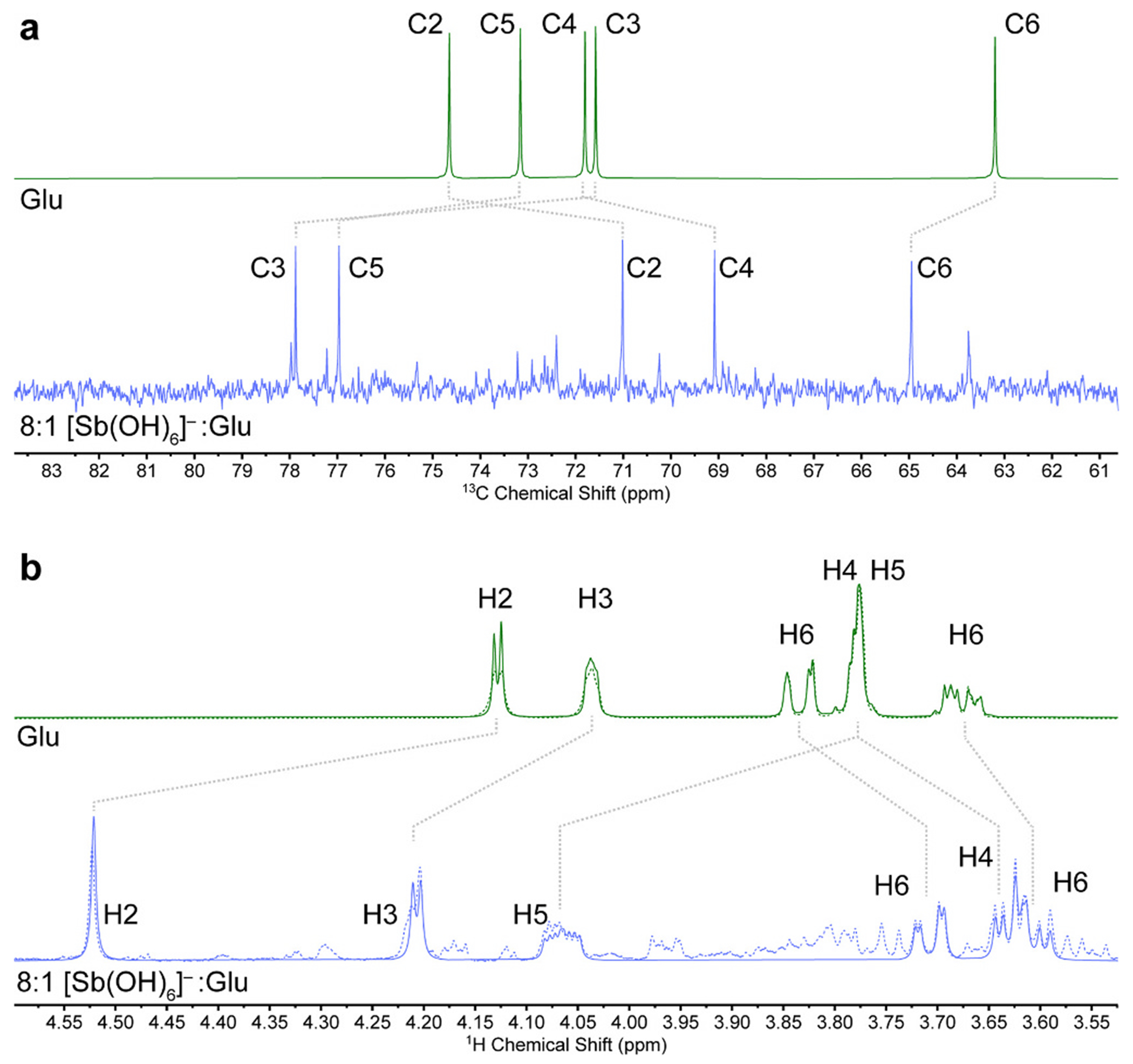
Stacked (a) ^13^C{^1^H} NMR spectra (125 MHz, D_2_O, pD 7) and (b) ^1^H NMR spectra (500 MHz, D_2_O, pD 7) of potassium gluconate (Glu, *top*) and an 8:1 mixture of K[Sb(OH)_6_] and potassium gluconate (*bottom*). In (b), the experimental spectra are shown as dashed lines and spectra simulated using the parameters in Table 1 are shown as solid lines.

With the assignments in hand, it was possible to assess which of the signals shifted upon complexation to Sb (Table 1, Fig. 4). The largest change among the ^13^C resonances is a 6.27 ppm downfield shift for C3. The corresponding H3 resonance similarly shifts downfield by 0.18 ppm. The C5 and H5 signals both exhibit large downfield shifts as well. In contrast, however, the C4 and H4 signals shift upfield. The C2 signal shifts upfield, but the corresponding H2 signal exhibits the largest downfield shift of all the protons. These results underscore the fact that caution needs to be exercised when drawing structural conclusions from such NMR data. In previous modelling work [25,26], we have observed that coordination of vicinal diols to an Sb(V) center can exhibit shifts that vary in potentially non-intuitive ways with the stereochemistry of the ligand.

Upon coordination of gluconate to Sb(V), the coupling constants for the signals also change. In the case of free gluconate, strong second-order effects limit the extent to which coupling constants can be read directly from the spectrum, but they could be obtained by spectral simulation (Fig. 4). Indeed, these simulations were needed to obtain the accurate chemical shifts for free gluconate that were used to calculate the Δδ and Δ*J* values described above. Binding to Sb(V) spreads out the 1H resonances substantially, permitting an initial first-order spectral analysis that served as a starting point for a full second-order simulation of the spectrum of the primary Sb-bound product (Table 1). Notably, ^3^*J*_4–5_ rose to 9.4 Hz. This increase could reflect a change in the torsion angle about C4–C5, such that H4 and H5 remain consistently anti to one another (or eclipsed). The values of ^3^*J*_5-6a_ and ^3^*J*_5-6b_ are consistent with a torsional setting about the C5–C6 bond that places H5 gauche to both H6_a_ and H6_b_, with H5–C5–C6–H6_b_ being smaller than H5–C5–C6–H6_a_. We have previously reported such an orientation for an Sb(V) complex of S-1,2-propanediolate [25]. Although a full structural determination is not currently possible on the basis of these data, we believe that this well-defined set of spectroscopic parameters will be invaluable in assessing the validity of future models of the structures of these drugs.

Although the species described above is the major product formed in the reaction of K[Sb(OH)_6_] and potassium gluconate, the ^1^H–^13^C HSQC, ^1^H–^1^H COSY, and ^1^H–^13^C HMBC spectra also permitted us to assign the signals of the next highest-population product, referred to hereafter as the secondary product (Table 2). The CH_2_ group (H6_a_′ and H6_b_′) could again be identified from the only set of negatively phased HSQC signals. A signal at 3.49 ppm exhibited COSY cross-peaks to both H6′ signals, thereby identifying it as H5′. H5′ in turn had a COSY cross-peak to one other signal at 3.75 ppm that was identified as H4′. The remaining two secondary product signals in the 1H NMR spectrum have chemical shifts of 4.67 ppm and 4.22 ppm. In the COSY spectrum, these signals coupled very weakly to one another but did not have cross-peaks to any other signal. In the HMBC spectrum, the ^13^C signal at 181.99 ppm corresponding to C1′ coupled strongly to the ^1^H signal at 4.22 ppm and weakly to the ^1^H signal at 4.67 ppm, suggesting that the former is H2′ and the latter is H3′. Simulation of the ^1^H NMR spectrum again permitted coupling constants to be estimated. There was an increase in ^3^*J*_4–5_ to 9 Hz similar to that observed for the primary product.

**Fig. 5.**
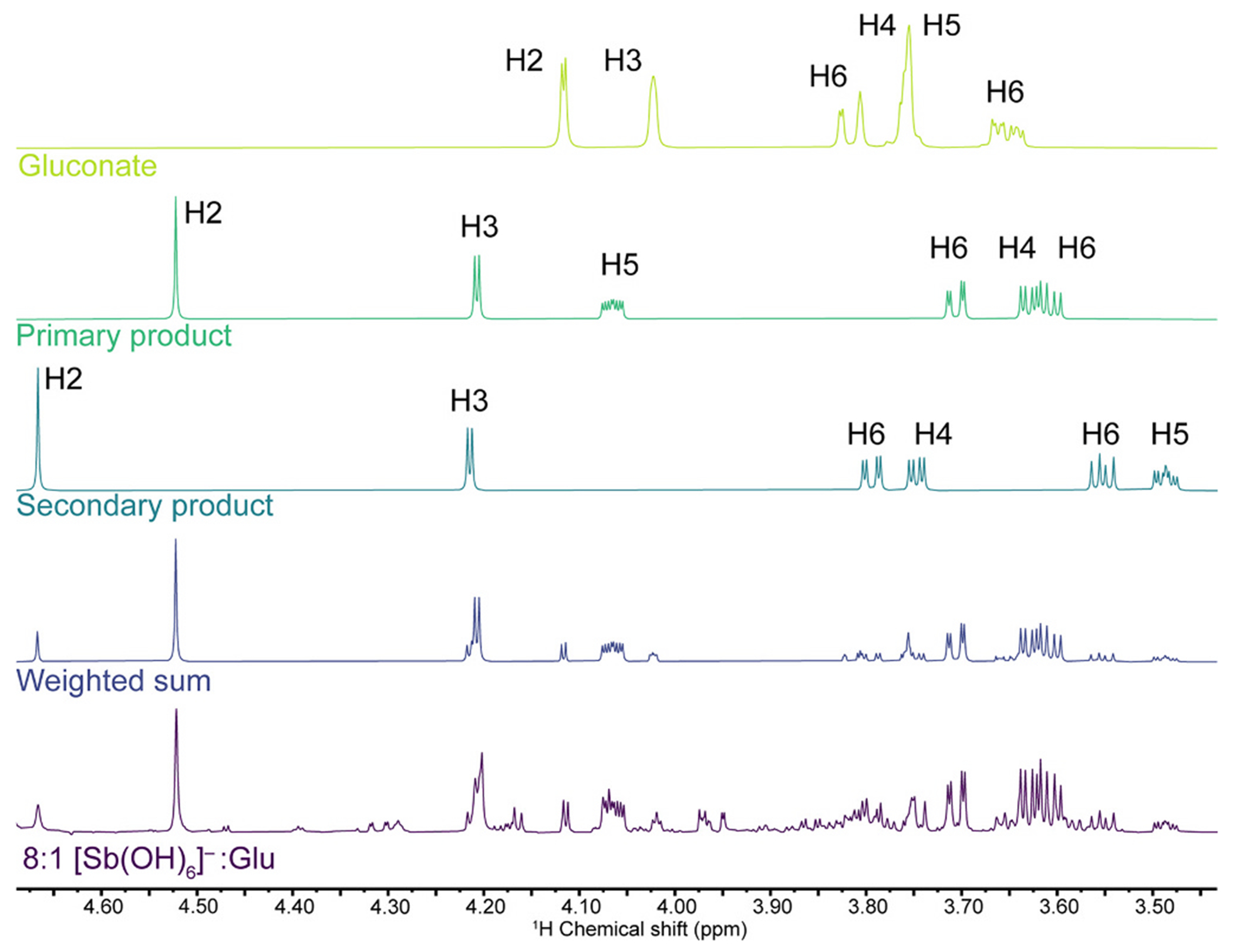
^1^H NMR spectrum (800 MHz, D_2_O, pD 7) of an 8:1 mixture of K[Sb(OH)_6_] stacked with simulations of the NMR spectra of gluconate, the primary product, and the secondary product, as well as a weighted sum of the three to simulate the experimental spectrum.

We next collected data from an 8:1 mixture of K[Sb(OH)_6_] and potassium gluconate in D_2_O (pD 7) at 800 MHz to better separate all of the signals. Using the spectroscopic parameters determined above, it was possible to simulate the spectrum of the reaction mixture (Fig. 5). The quality of the simulation at the different field strength provides further support for the numerical simulation parameters. The spectrum is pre-dominantly composed of the primary product, the secondary product, and residual free gluconate in a 100:35:25 ratio. There appears to be at least one additional gluconate-based product, but severe overlap prevents a full assignment of this species. From the integrated areas of putative signals that are observed for it, this species is present in even lesser amounts.

### 3.4. Reaction of Na[Sb(OH)_6_] with potassium gluconate

The results in Sections 3.1–3.3 were obtained by preparing homogeneous solutions of the potassium salts of [Sb(OH)_6_]^−^ and gluconate. The solubility of Na[Sb(OH)_6_] is too low to perform the full range of analogous experiments with the sodium salts. If a homogeneous 50 mM solution of potassium gluconate was combined with an equimolar amount of Na[Sb(OH)_6_] (analogous to the first reaction described in Section 3.2), the solid Na[Sb(OH)_6_] did not fully dissolve. Even after heating at 70 °C for 2 h, the colorless solid remained undissolved. If, however, Na[Sb(OH)_6_] was combined with a six-fold excess of potassium gluconate, the reaction mixture became homogeneous within 30 min. A ^13^C{^1^H} NMR spectrum of this solution was collected after heating at 70 °C for 2 h. Prominent free gluconate signals were observed, consistent with the presence of excess gluconate, but an additional set of signals was also present that agreed well with those of the predominant product formed in the reaction between potassium gluconate and K[Sb (OH)_6_] (Fig. S11).

### 3.5. Comparison to authentic sodium stibogluconate and Pentostam^®^

The reactions described above align with the procedures used to prepare stibogluconate in research laboratories [17]. To ensure that the results are relevant to the clinically employed drugs, we compared these results to spectra collected from a solution prepared by dissolving solid sodium stibogluconate (supplied by Sigma Aldrich) in D_2_O, as well as a clinical sample of Pentostam^®^ (manufactured by GSK). Because Pentostam^®^ is formulated as an aqueous solution, we chose to compare these materials using ^13^C{^1^H} NMR spectroscopy.

**Fig. 6.**
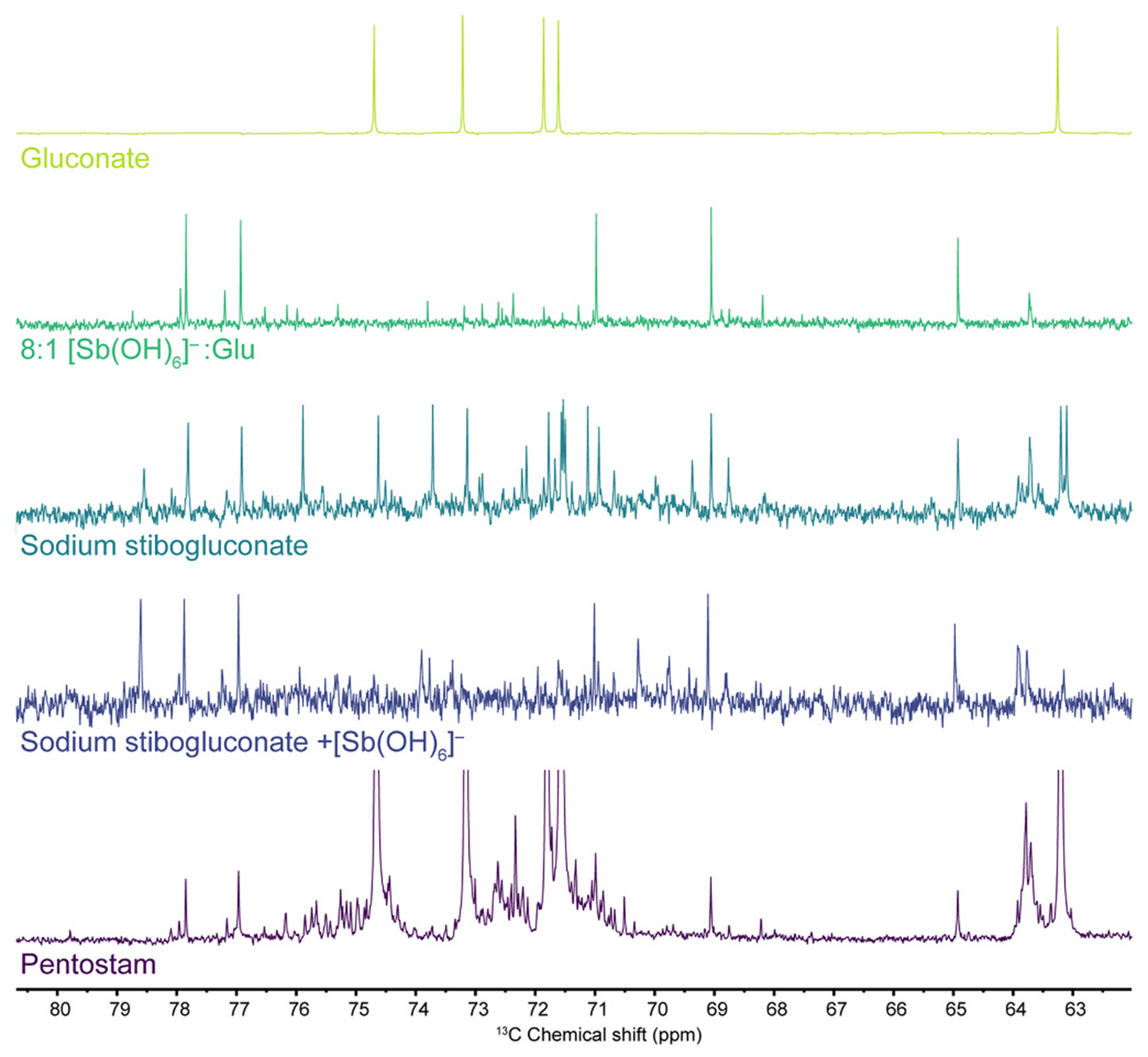
^13^C{^1^H} NMR spectra (D_2_O, pD 7) of potassium gluconate, an 8:1 mixture of K[Sb(OH)_6_] and potassium gluconate, commercial sodium stibogluconate, and commercial sodium stibogluconate with 1.3 mass equivalents of K[Sb(OH)_6_]. The bottom spectrum is that of a clinical sample of Pentostam^®^ spiked with 10% D_2_O. It is vertically scaled to visualize the signals other than those of free gluconate.

The solution of solid sodium stibogluconate in D_2_O shows all of the previously identified signals corresponding to the major and minor products of the reaction of [Sb(OH)_6_]^−^ and gluconate (Fig. 6). There are also prominent signals corresponding to free gluconate, which stands to reason given that an excess of gluconate is needed to ensure that the Sb (V) is not simply precipitated as Na[Sb(OH)_6_] (see Section 3.4). An additional set of as-yet-unassigned gluconate-based signals was also present with aliphatic resonances at 78.6, 75.94, 73.9, 70.3, and 63.9 ppm. As with the controlled reaction mixtures of K[Sb(OH)_6_] and potassium gluconate, addition of extra K[Sb(OH)_6_] to the solution of sodium stibogluconate results in a decrease in the relative intensity of signals other than those of the primary product assigned above, including those of the unidentified product noted above. Additional minor signals are present, including those of the secondary product assigned above.

A clinical preparation of Pentostam^®^ was obtained from the US Centers of Disease Control and Prevention, spiked with 10% D_2_O for locking and shimming, and analyzed by ^13^C{^1^H} NMR spectroscopy. The most prominent signals in the spectrum arise from excess free gluconate (Fig. 6). The next most prominent set of signals corresponds to the primary product of the reaction between [Sb(OH)_6_]^−^ and gluconate. A number of weaker signals are present between 70 and 76 ppm. A prominent cluster of signals near 63.8 ppm was also present, as was the case for the solution of sodium stibogluconate.

### 3.6. Stibogluconate products are not observed by ^121^Sb NMR spectroscopy

^121^Sb{^1^H} NMR spectra collected from the reactions of 1:1 potassium gluconate and K[Sb(OH)_6_] (50 mM initial concentrations of each) showed signals for the unreacted [Sb(OH)_6_]^−^ at 296 ppm (Fig. S12). In the same way that excess [Sb(OH)_6_]^−^ led to complete consumption of gluconate (*vide supra*), reaction of K[Sb(OH)_6_] (50 mM initial concentration) with 10 equiv of potassium gluconate showed complete consumption of K[Sb(OH)_6_], as assessed by the disappearance of the ^121^Sb NMR signal at 296 ppm (Fig. S13). Unlike the ^1^H NMR data, however, no new signals for Sb-containing products appeared in the ^121^Sb NMR spectrum. We note that for compounds with symmetry even moderately reduced from octahedral or tetrahedral, the large quadrupole moment of the *I* = 5/2 ^121^Sb nucleus typically results in rapid relaxation and signals often too broad to observe. As expected, the rigorously Oh-symmetric [SbCl_6_]^−^ has a sharp signal (FWHM = 270 Hz for (PPh_4_)[SbCl_6_] in DMF). As such, it is perhaps surprising that the nominally octahedral [Sb (OH)_6_]^−^ anion has a much broader signal (FWHM = 2400 Hz for K[Sb (OH)_6_] in D_2_O) (Fig. S14). We confirmed that, in the solid state, the [Sb (OH)_6_]^−^ anion does indeed have approximately octahedral symmetry (Fig. S18) and we suspect that the breadth of the [Sb(OH)_6_]^−^^121^Sb NMR signal arises from fluctuating H-bonding with the D_2_O solvent. Given that even nominally octahedral complexes can have drastically broad-ened signals, we propose that the lack of ^121^Sb NMR signals for any of the products of the reaction of gluconate with [Sb(OH)_6_]^−^ arises from rapid quadrupolar relaxation facilitated by their lowered symmetry.

### 3.7. [Sb(OH)_6_]^−^ can be quantified by ^121^Sb NMR spectroscopy

Although the signals from [Sb(OH)_6_]^−^ are broad, we did observe that they systematically varied in intensity in a reliable fashion. To the best of our knowledge, ^121^Sb NMR spectroscopy has not been used as an analytical technique to quantify the concentrations of Sb-containing species in solution. Given that we ultimately aimed to probe the concentrations of Sb complexes engaged in a dynamic equilibrium, we elected to perform our quantitation using a standard solution in a coaxial tube. The physical separation of the analyte and standard solutions prevents the standard from interfering with the equilibrium. A series of D_2_O solutions of K[Sb(OH)_6_] afforded a linear calibration curve over the concentration range 3.8–68.5 mM (Figs. S14, S15).

### 3.8. Thermodynamic stability of primary product formed between [Sb (OH)_6_]^−^ and gluconate

**Fig. 7.**
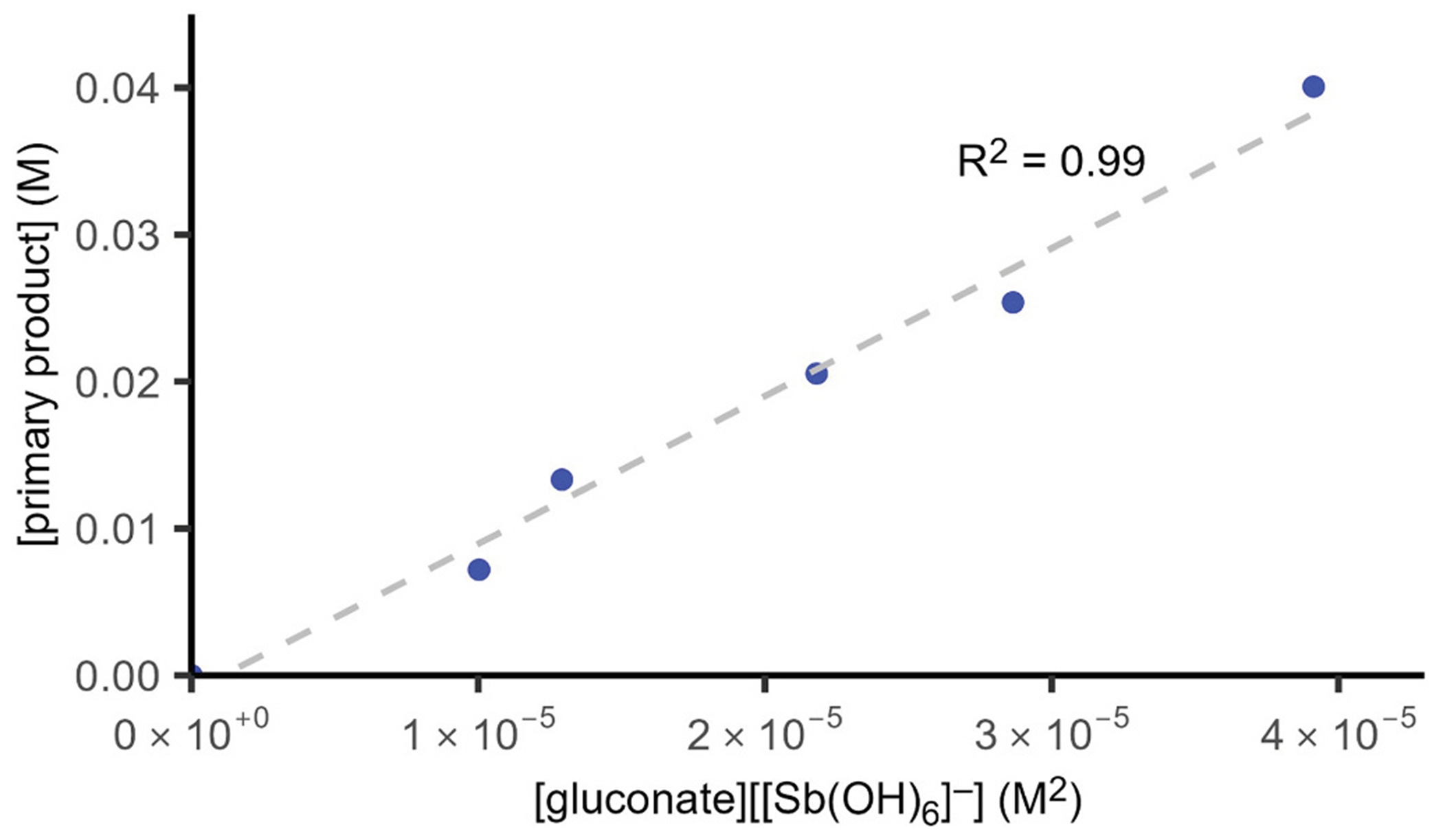
Determination of the equilibrium constant for the reaction between K [Sb(OH)_6_] and potassium gluconate to give the primary product described in this paper (*K* = 1006 M^−1^).

With our capacity to quantify [Sb(OH)_6_]^−^, however, we also had the capacity to quantify the stability constant of the primary, putatively 1:1 product. As the overall concentration of the mixture was increased, ^1^H NMR experiments clearly showed that the equilibrium shifted toward complexation of Sb(V) by gluconate, and quantification of the concentrations of unbound gluconate, unligated [Sb(OH)_6_]^−^, and primary 1:1 product allowed us to determine the stability constant to be 1006 M^−1^ (Figs. 7, S16, S17). This equilibrium behavior may explain the need for an excess of free gluconate in commercial and clinical preparations of sodium stibogluconate because such excess levels are needed to ensure effectively complete complexation of the Sb(V).

## 4. Conclusion

As one of the two pentavalent antimonials, sodium stibogluconate remains a frontline therapy for various forms of leishmaniasis in multiple countries. Despite its widespread use, the molecular structure of this drug remains unknown. The NMR spectroscopic data presented here suggest that stibogluconate contains one predominant species, the NMR spectroscopic signals of which have been fully assigned. Work is ongoing to use these spectroscopic assignments in conjunction with other experimental techniques to elucidate the molecular structure of the primary Sb-containing species in this drug and ultimately use that structural information to better understand and improve antileishmanial therapy.

## Supplementary Material

Supporting Information

## Figures and Tables

**Table 1:** NMR spectroscopic parameters for the major product of the reaction between potassium gluconate and K[Sb(OH)_6_].

	^1^H				^13^C	
Atom (H or C)	δ (ppm)	Δδ ^a^ (ppm)	*J* (Hz)	Δ*J* ^b^ (Hz)	δ (ppm)	Δδ ^b^ (ppm)
1	–	–	–	–	182.88	−3.66
2	4.52	−0.41	^3^*J*_2–3_ ≈ 0 ^d^	2.0	70.98	3.67
3	4.21	−0.18	^3^*J*_3–4_ = 3.8	−2.3	77.85	−6.27
4	3.63	0.13	^3^*J*_4–5_ = 9.4	−2.4	69.05	2.75
5	4.07	−0.31	^3^*J*_5-6a_ =	−0.5	76.93	−3.77
			2.5	0.8		
			^3^*J*_5-6b_ =			
			5.2			
6a ^c^	3.71	0.11	^2^*J*_6a-6b_ =	1.6	64.92	−1.72
			−11.6			
6b ^c^	3.61	0.05	–			

a Δδ = δ_free gluconate_ − δ_bound gluconate_.

b Δ|*J*| = *J*_free gluconate_ − *J*_bound gluconate_.

c The 6_a_ proton is consistently assigned as that which is most downfield. We cannot rule out the possibility that the 6_a_ and 6_b_ protons change with regards to which is more downfield upon complexation with Sb.

d A very weak COSY cross-peak is observed (Fig. 3); the simulations were performed with a coupling constant of 0 Hz.

**Table 2:** NMR spectroscopic parameters for the secondary product of the reaction between potassium gluconate and K[Sb(OH)_6_].

	^1^H				^13^C	
Atom (H or C)	δ (ppm)	Δδ ^a^ (ppm)	*J* ^b^ (Hz)	Δ*J* ^b^ (Hz)	δ (ppm)	Δδ ^b^ (ppm)
1′	–	–	–	–	181.99	−2.78
2′	4.22	−0.19	^3^*J*_2–3_ = 0	−2.0	68.19	−3.29
3′	4.67	−0.55	^3^*J*_3–4_ = 3.8	−2.3	77.94	3.38
4′	3.75	0.01	^3^*J*_4–5_ = 9.0	−2.0	77.20	−5.39
5′	3.49	0.27	^3^*J*_5-6a_ =	−1.1	72.37	0.79
			3.1	−0.9		
			^3^*J*_5-6b_ =			
			6.9			
6_a_′ ^c^	3.79	0.02	^2^*J*_6a-6b_ =	1.8	63.73	−0.54
			−11.8			
6_b_′ ^c^	3.55	0.10				

a Δδ = δ_free gluconate_ − δ_bound gluconate_.

b Δ|*J*| = |*J*|_free gluconate_ − |*J*|_bound gluconate_.

c The 6_a_′ proton is consistently assigned as that which is most downfield. We cannot rule out the possibility that the 6_a_′ and 6_b_′ protons change with regards to which is more downfield upon complexation with Sb.

## Data Availability

Data are provided within the manuscript or supplementary information files. Raw data sets are available upon request. Crystallographic data for K[Sb(OH)_6_] and (PPh_4_)[SbCl_6_] have been deposited at the Cambridge Crystallographic Data Centre, under deposition numbers CCDC 2387910–2387911, and can be obtained from https://www.ccdc.cam.ac.uk/structures/.

## Appendix A. Supplementary data

Supplementary data to this article can be found online at https://doi.org/10.1016/j.jinorgbio.2026.113237.
