# Supplementary material for: NMR spectroscopic evidence that the antileishmanial drug sodium stibogluconate comprises one predominant molecular species
Source: J Inorg Biochem. Author manuscript; Available in PMC 2026 Apr 14. (PMC13078173; doi:10.1016/j.jinorgbio.2026.113237)
Supplement: Supporting Information [file NIHMS2157996-supplement-Supporting_Information.pdf]

Supplementary data for

NMR Spectroscopic Evidence that the Antileishmanial Drug Sodium Stibogluconate Comprises One Predominant Molecular Species

Alissa Lance-Byrne[a], Juliet C. Gee[b], and Timothy C. Johnstone[b]*

[a] Molecular, Cellular, and Developmental Biology, University of California Santa Cruz, Santa Cruz, California 95064, United States.

[b] Department of Chemistry and Biochemistry, University of California Santa Cruz, Santa Cruz, California 95064, United States.

Correspondence: johnstone@ucsc.edu

CONTENTS

	Page
Figures S1-S2: ESI mass spectra	S3
Figures S3-S17: NMR spectra	S5
Figures S18-S19: Thermal ellipsoid plots of crystallographic data	S15
Table S1: Crystallographic parameters	S16
References	S16

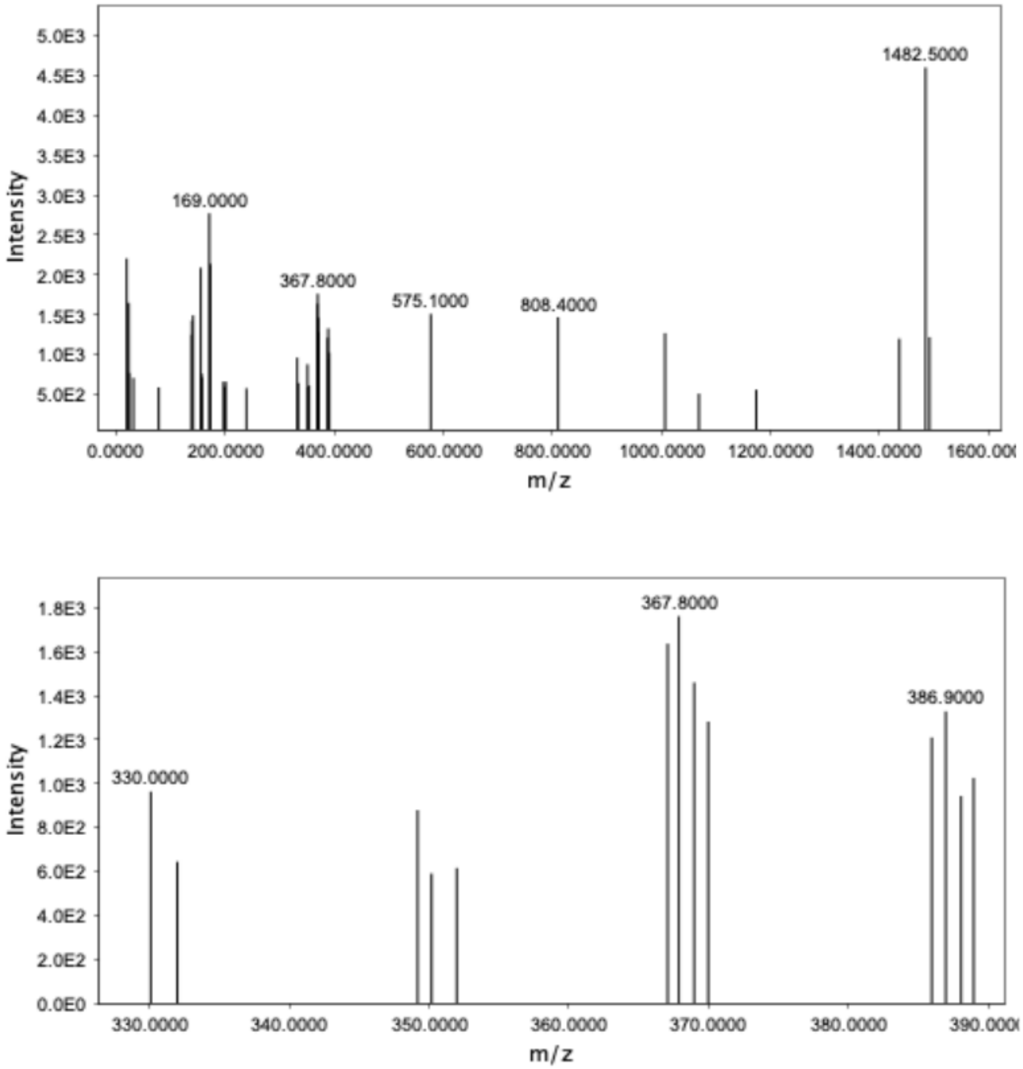

Figure S1. *Top:* Mass spectrum (ESI-MS, negative mode) of a 1:1 aqueous solution of K[Sb(OH)$_6$] and potassium gluconate (0.25 mM each) that was heated at 70 °C for 2 h. *Bottom:* Expanded region of interest. Signal positions match those previously reported [1].

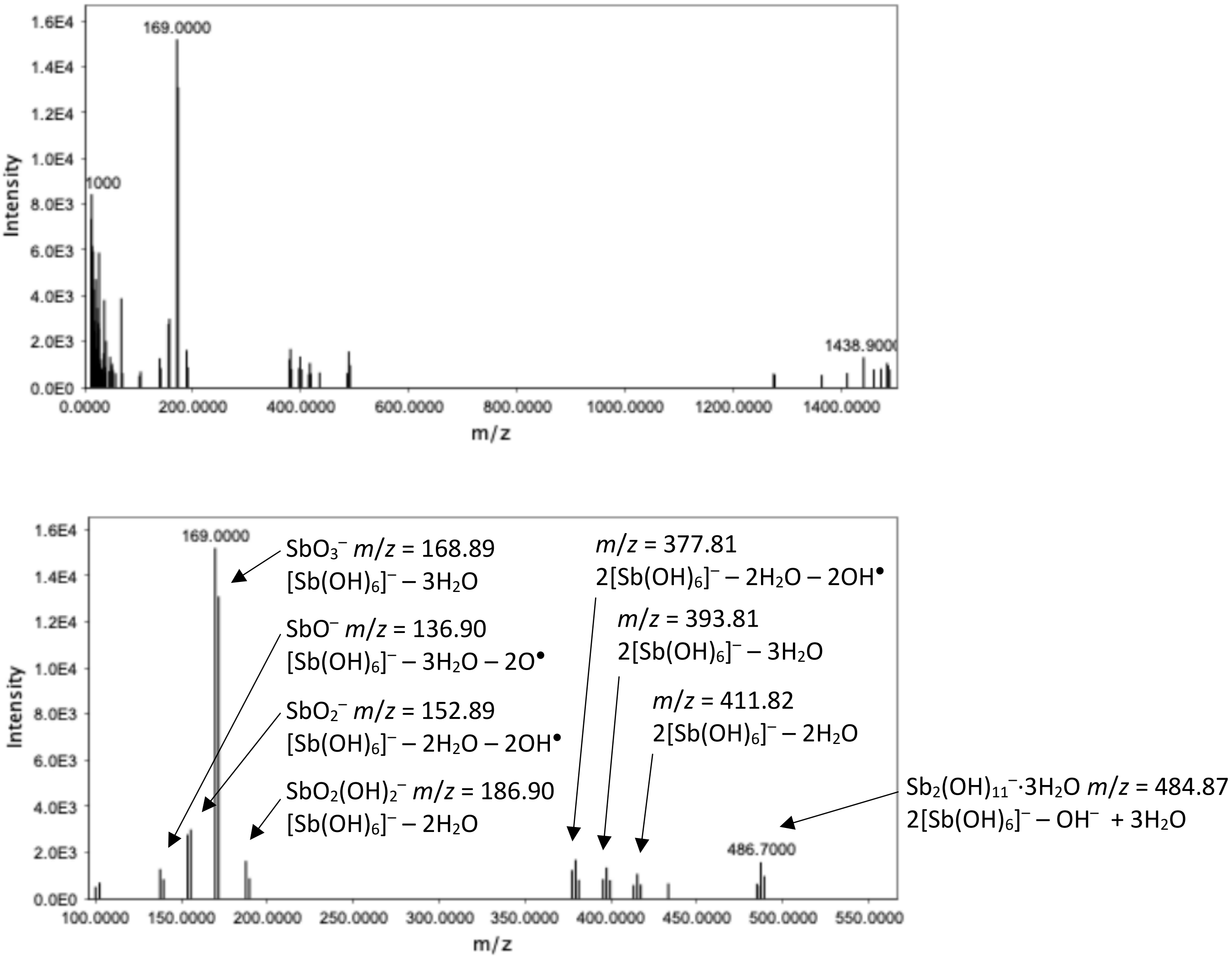

Figure S2. *Top:* Mass spectrum (ESI-MS, negative mode) of an aqueous solution of K[$Sb(OH)_6$] (0.25 mM). [$Sb(OH)_6$]$^-$ would have a doublet signal at *m/z* = 222.92 and 224.92. Bottom: Expanded region of interest.

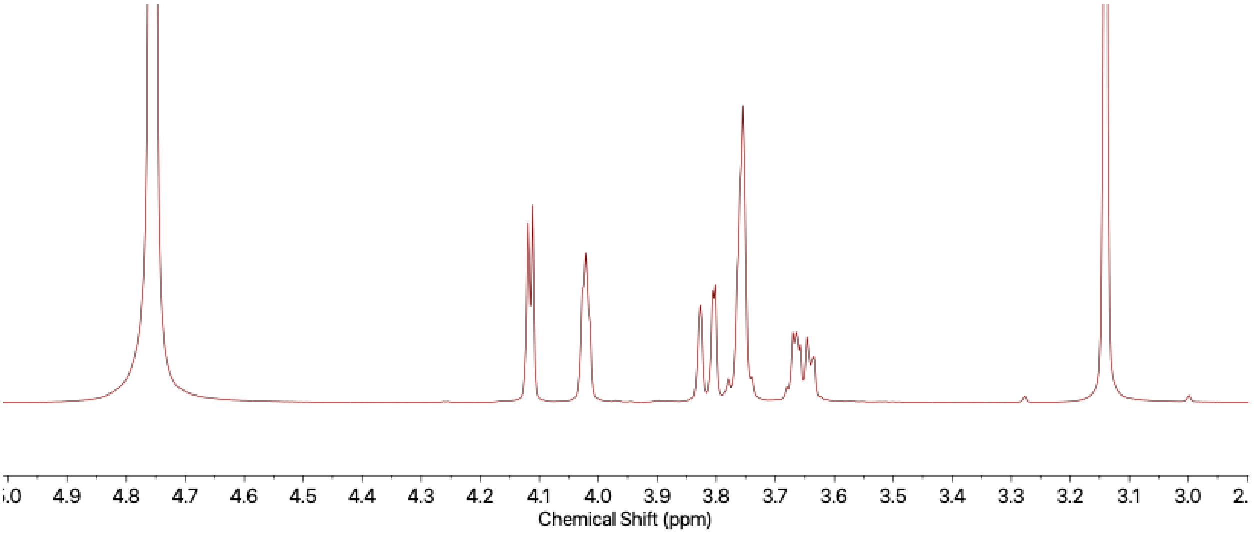

Figure S3. ^{1}H NMR spectrum (D_2O, 500 MHz, pD 7) of a 50 mM solution of potassium gluconate.

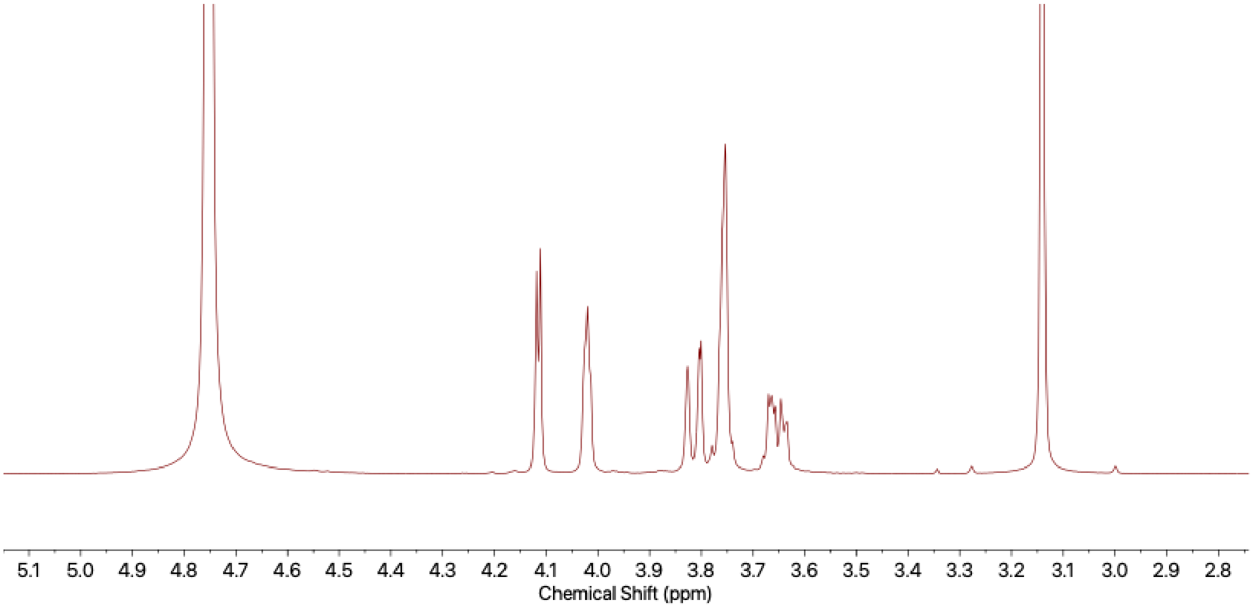

Figure S4. ^{1}H NMR spectrum (D_2O, 500 MHz, pD 7) of a 50 mM solution of potassium gluconate combined 1:1 with $K[Sb(OH)_6]$ collected < 10 min after mixing.

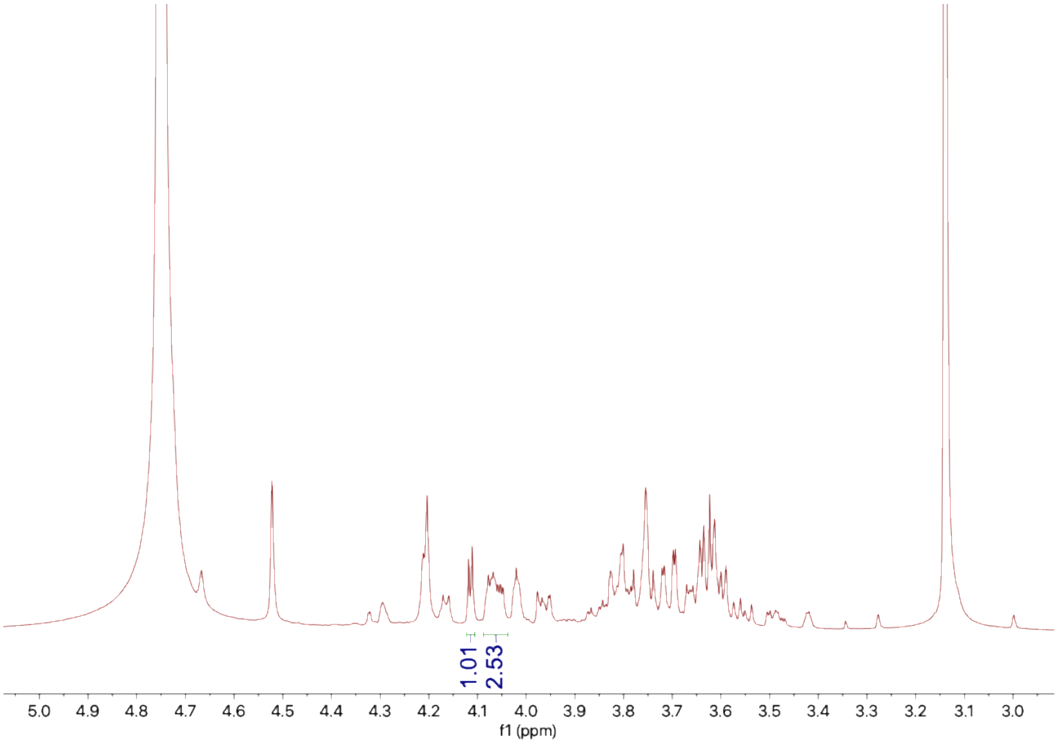

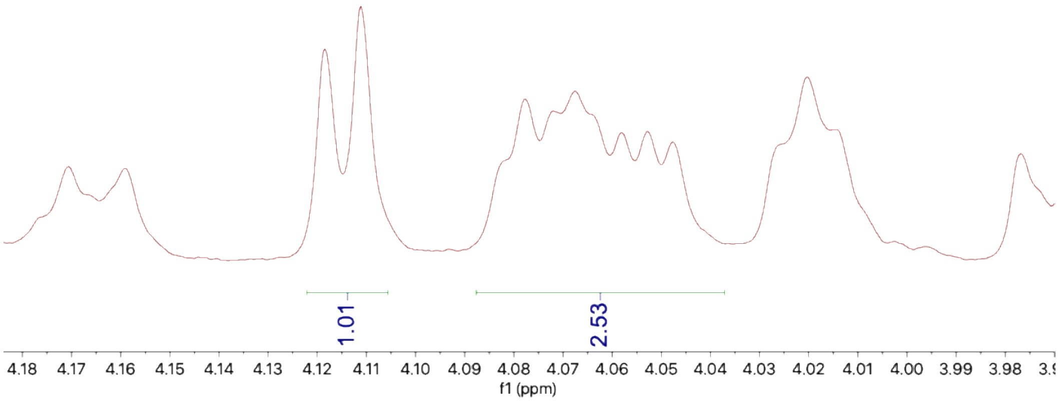

Figure S5. ^1H NMR spectrum (D_2O, 500 MHz, pD 7) of a 50 mM solution of potassium gluconate combined 1:1 with $K[Sb(OH)_6]$ collected < 10 min after heating for 2 h at 70 °C. *Top*: full-width spectrum. *Bottom*: expanded region of interest.

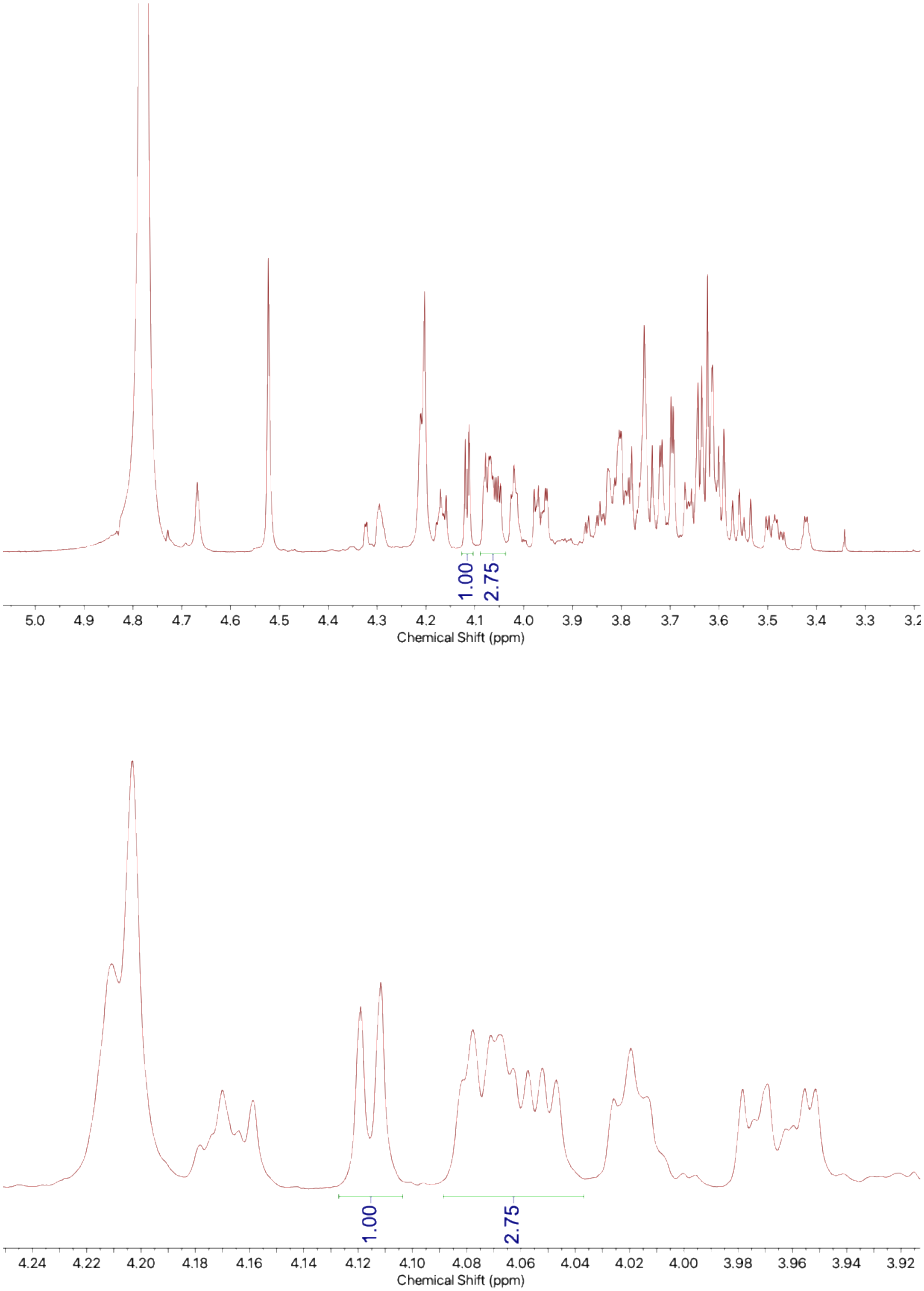

Figure S6. ^{1}H NMR spectrum (D_2O, 500 MHz, pD 7) of a 50 mM solution of potassium gluconate combined 1:1 with potassium $K[Sb(OH)_6]$ collected after heating for 2 h at 70 °C and subsequently incubating for 24 h at room temperature. *Top*: full-width spectrum. *Bottom*: expanded region of interest.

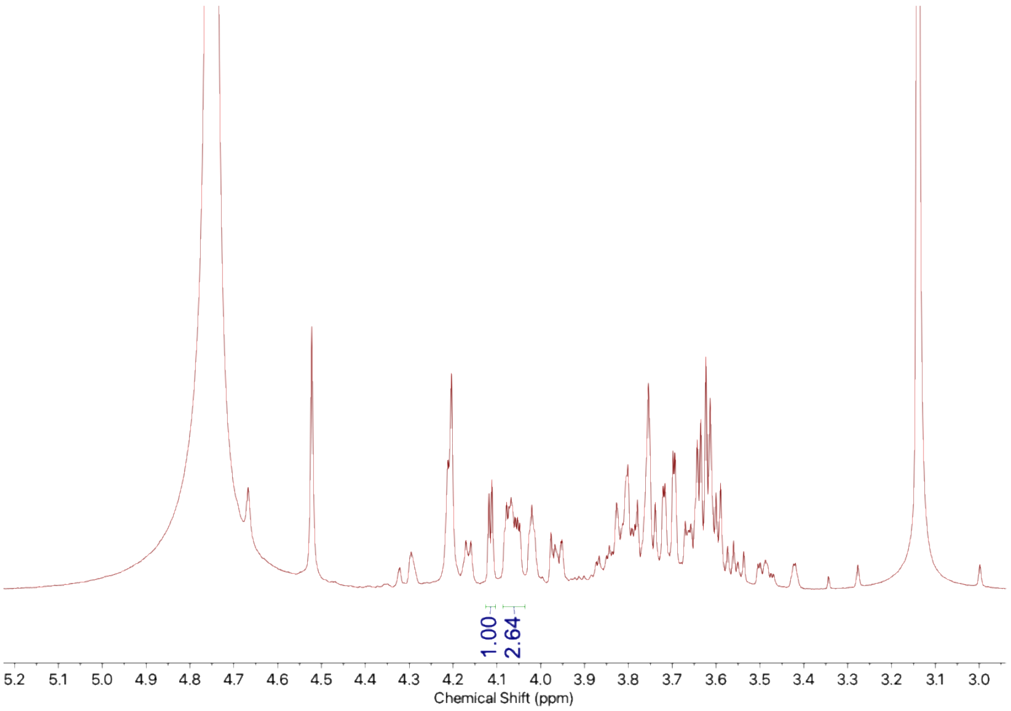

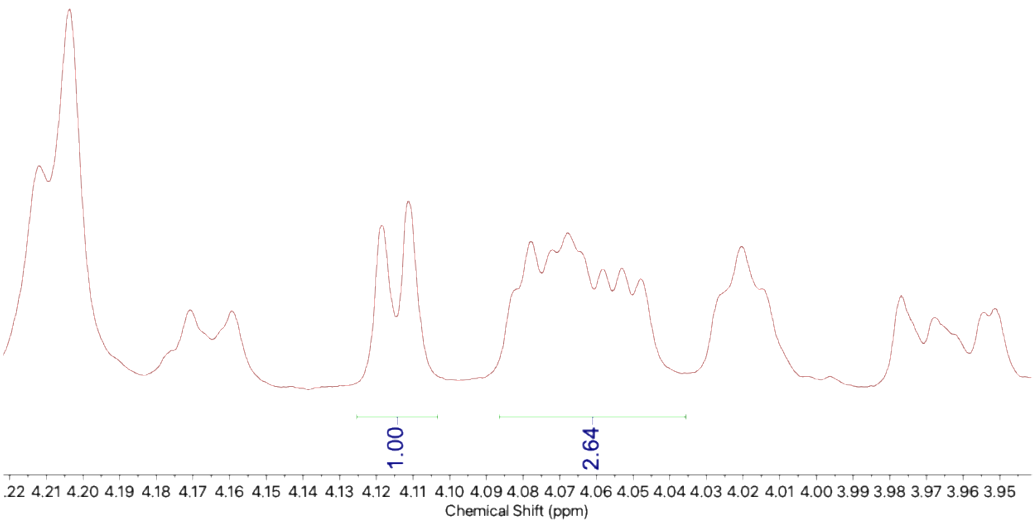

Figure S7. ^{1}H NMR spectrum (D_2O, 500 MHz, pD 7) of a 50 mM solution of potassium gluconate combined 1:1 with $K[Sb(OH)_6]$ collected < 10 min after heating for 24 h at 70 °C. *Top*: full-width spectrum. *Bottom*: expanded region of interest.

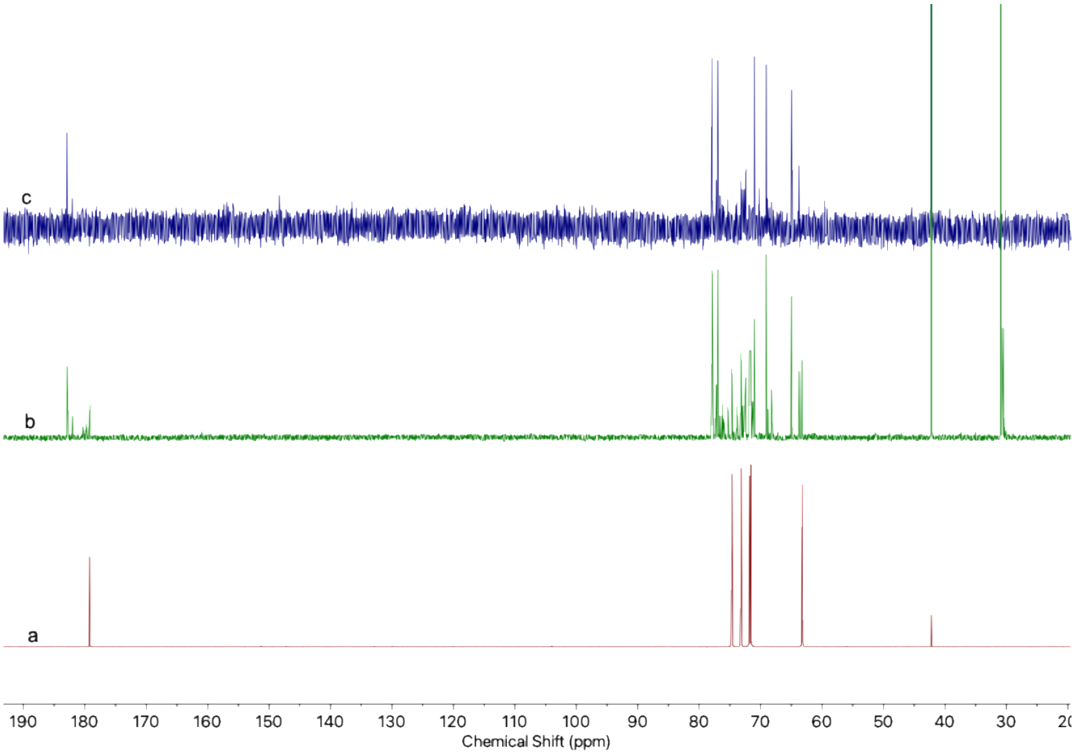

Figure S8. Full-width $^{13}C\{^1H\}$ NMR spectra (D_2O, 125 MHz, pD 7) of (a) 50 mM potassium gluconate, (b) a 50 mM solution of potassium gluconate reacted in a 1:1 ratio with $K[Sb(OH)_6]$, and (c) a 25 mM solution of potassium gluconate reacted with 8 equiv $K[Sb(OH)_6]$.

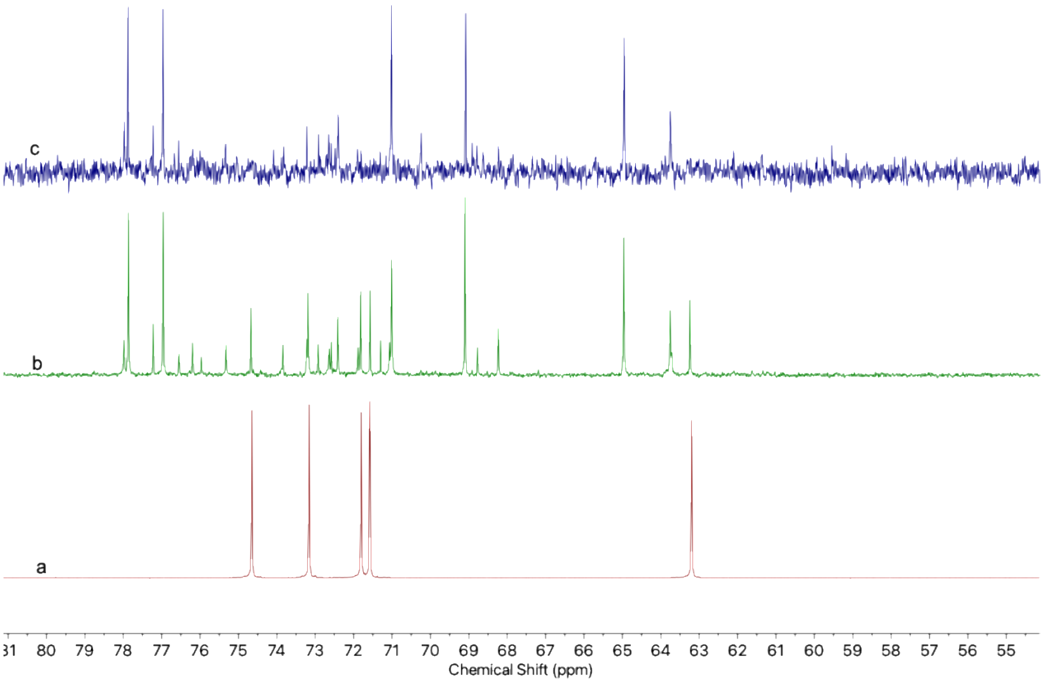

Figure S9. Expanded $^{13}C\{^1H\}$ NMR spectra (D_2O, 125 MHz, pD 7) of (a) 50 mM potassium gluconate, (b) a 50 mM solution of potassium gluconate reacted in a 1:1 ratio with $K[Sb(OH)_6]$, and (c) a 25 mM solution of potassium gluconate reacted with 8 equiv $K[Sb(OH)_6]$.

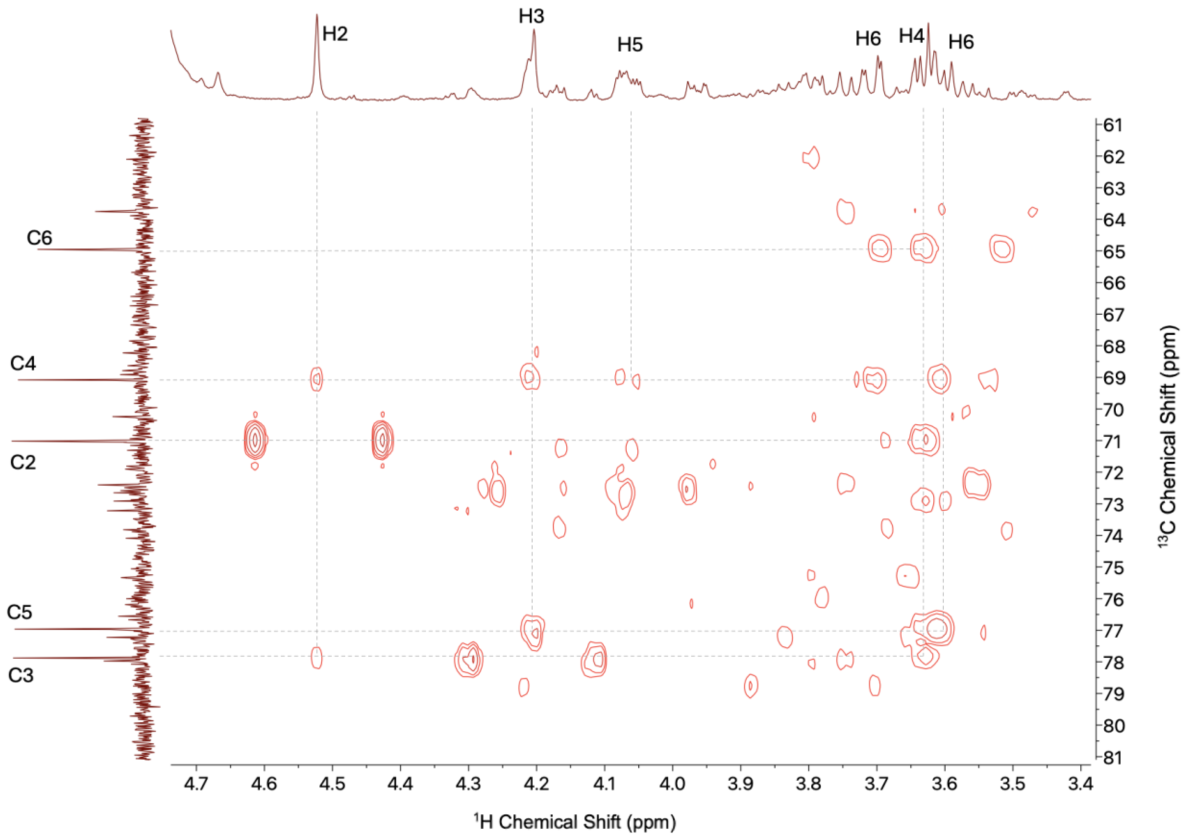

Figure S10. ^{1}H-^{13}C HMBC NMR spectrum (D_2O, 800 MHz, pD 7) of a 25 mM solution of potassium gluconate reacted with 8 equiv $K[Sb(OH)_6]$.

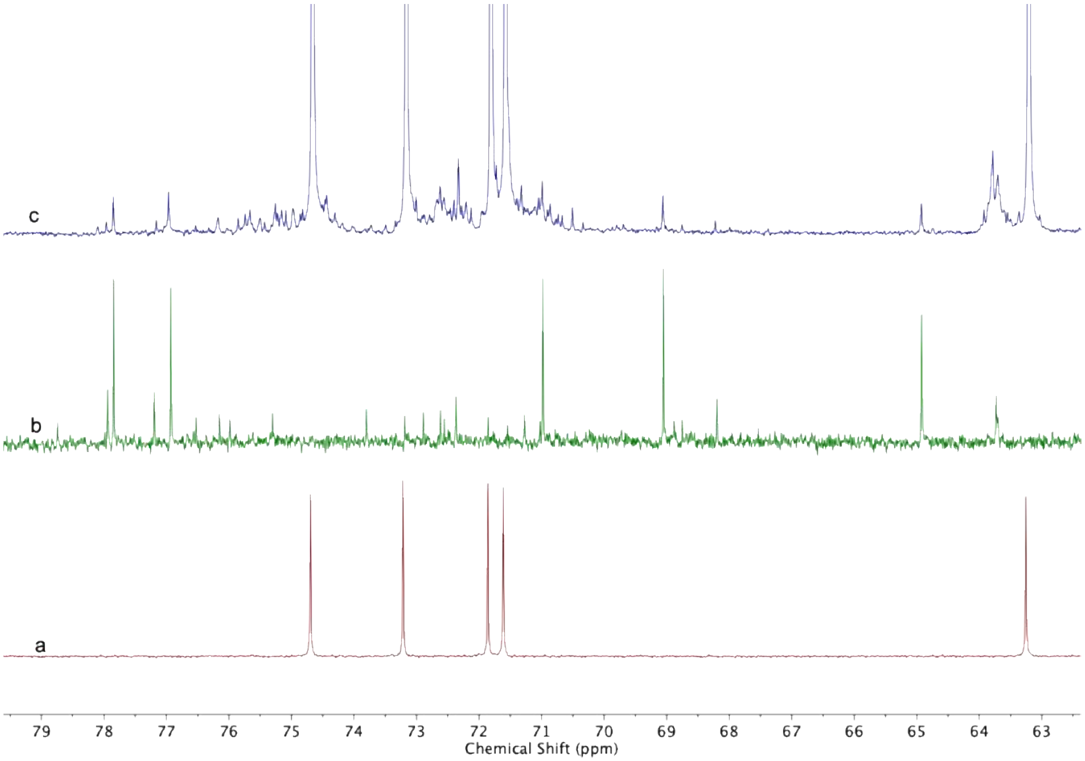

Figure S11. Expanded $^{13}C\{^{1}H\}$ NMR spectra of (a) potassium gluconate, (b) 25 mM potassium gluconate reacted 8:1 with $K[Sb(OH)_6]$, and (c) 50 mM $Na[Sb(OH)_6]$ reacted with a six-fold excess of potassium gluconate.

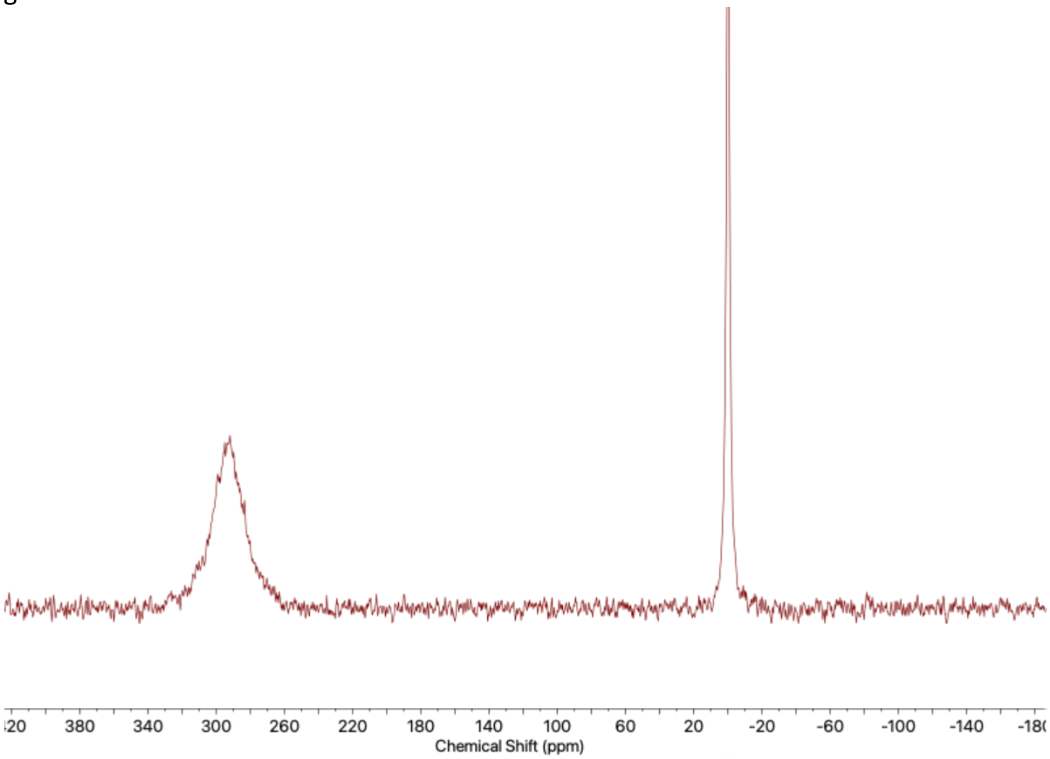

Figure S12. $^{121}Sb\{^{1}H\}$ spectrum (D_2O, 120 MHz, pD 7) of a 50 mM solution of potassium gluconate combined 1:1 with $K[Sb(OH)_6]$ collected < 10 min after heating for 2 h at 70 °C. The reference signal at 0 ppm is $(PPh_4)[SbCl_6]$ in DMF (collected using a coaxial insert).

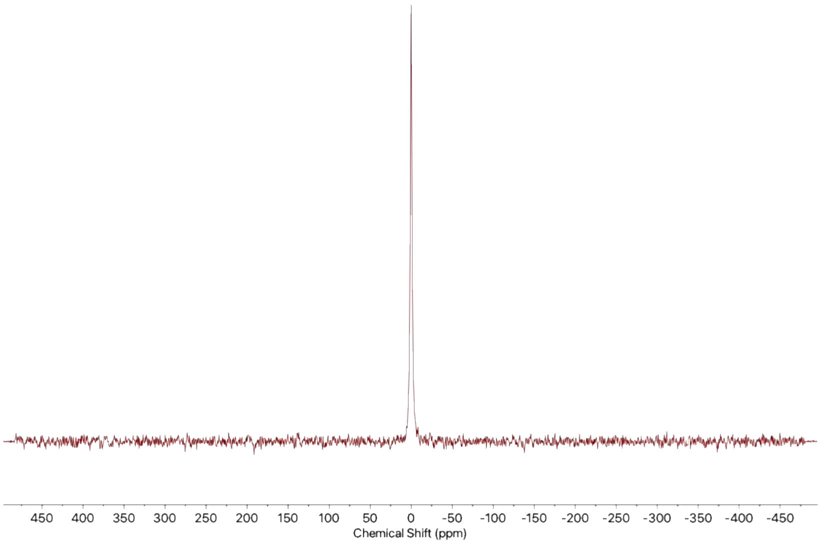

Figure S13. ${}^{121}Sb\{{}^{1}H\}$ NMR spectrum (D_2O, 120 MHz, pD 7) of 50 mM $K[Sb(OH)_6]$ combined with 10 equiv of potassium gluconate collected < 10 min after heating for 2 h at 70 °C. There is an absence of any signal other than the standard (DMF solution of $(PPh_4)[SbCl_6]$ in a coaxial insert) at 0 ppm.

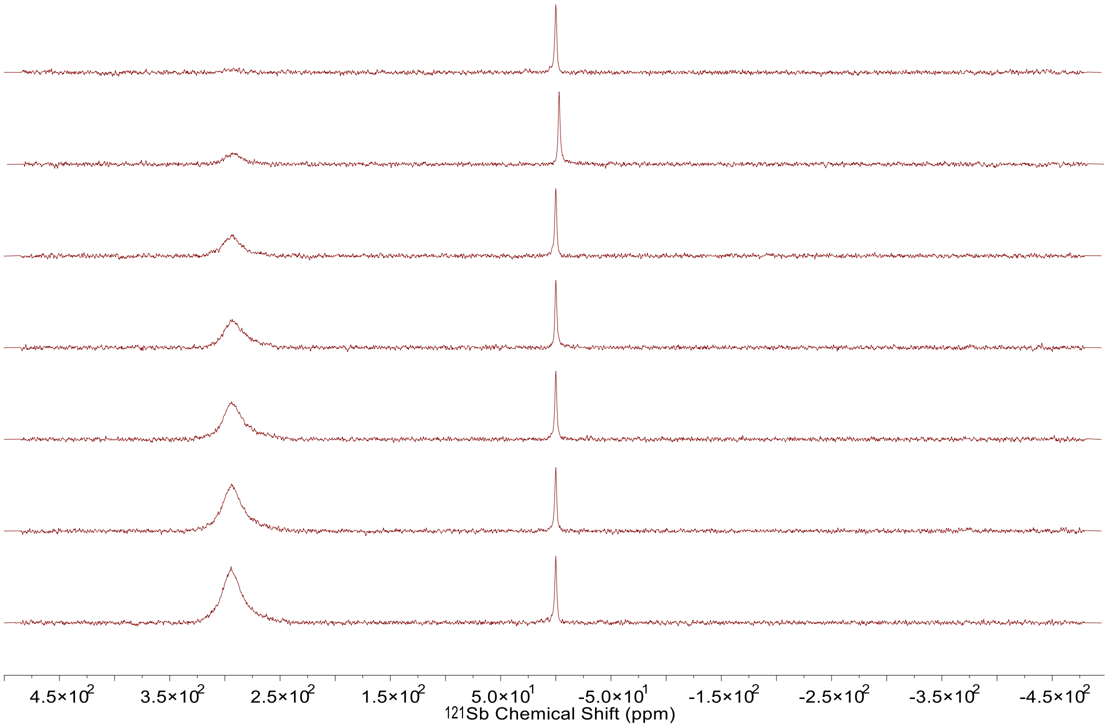

Figure S14. ${}^{121}Sb\{{}^{1}H\}$ NMR (D_2O, 120 MHz, pD 7) spectra of $K[Sb(OH)_6]$ and $(PPh_4)[SbCl_6]$ in DMF used to prepare the calibration curve for $K[Sb(OH)_6]$ quantification. $K[Sb(OH)_6]$ concentrations from top to bottom: 3.8, 11.4, 22.8, 34.2, 45.6, 57.1, 68.5 mM.

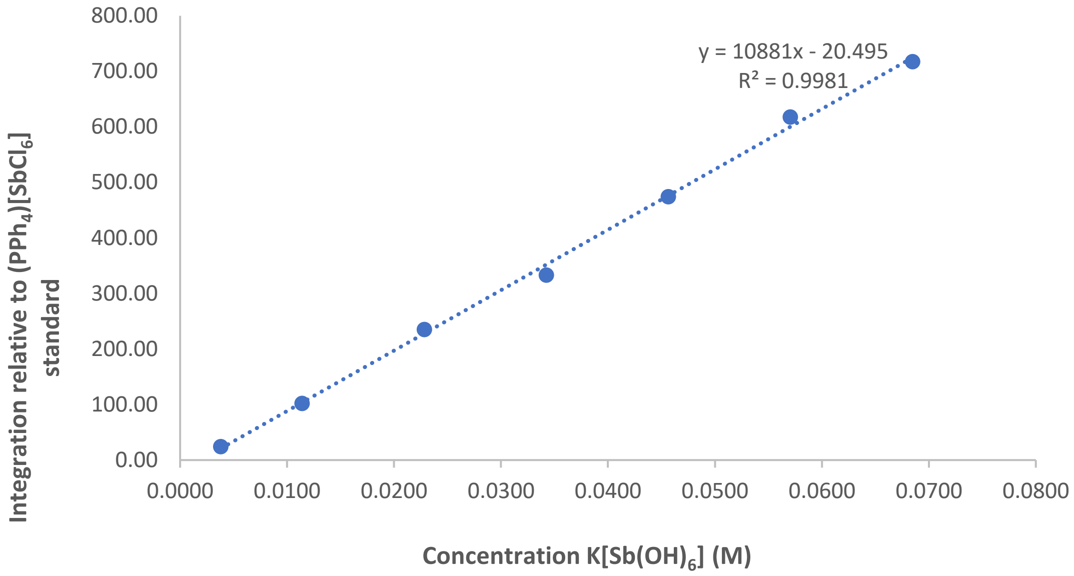

Figure S15. Calibration curve showing integration of $^{121}Sb\{^1H\}$ NMR resonances of $K[Sb(OH)_6]$ relative to those of a $(PPh_4)[SbCl_6]$ reference standard as a function of $K[Sb(OH)_6]$ concentration.

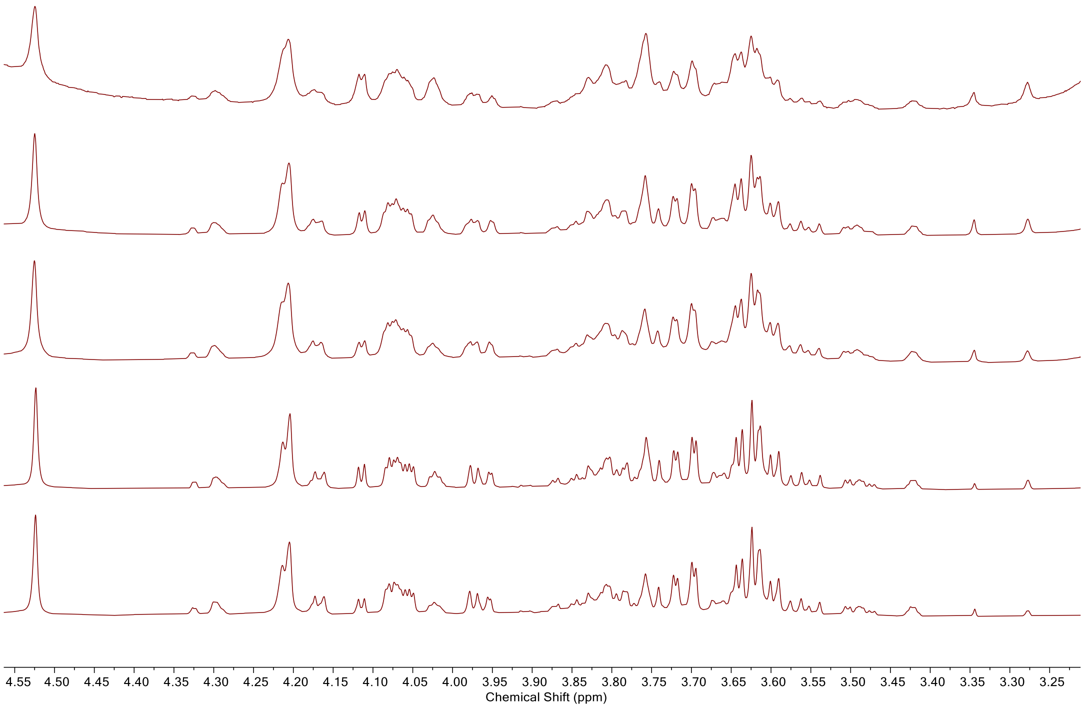

Figure S16. ^1H NMR spectra (D_2O, 500 MHz, pD 7) for the quantification of the equilibrium constant for the reaction between $K[Sb(OH)_6]$ and potassium gluconate to produce the primary product. All solutions contained $K[Sb(OH)_6]$ and potassium gluconate in a 1:1 molar ratio. From top to bottom: initial $K[Sb(OH)_6]$ concentration of 15, 25, 35, 45, 55 mM.

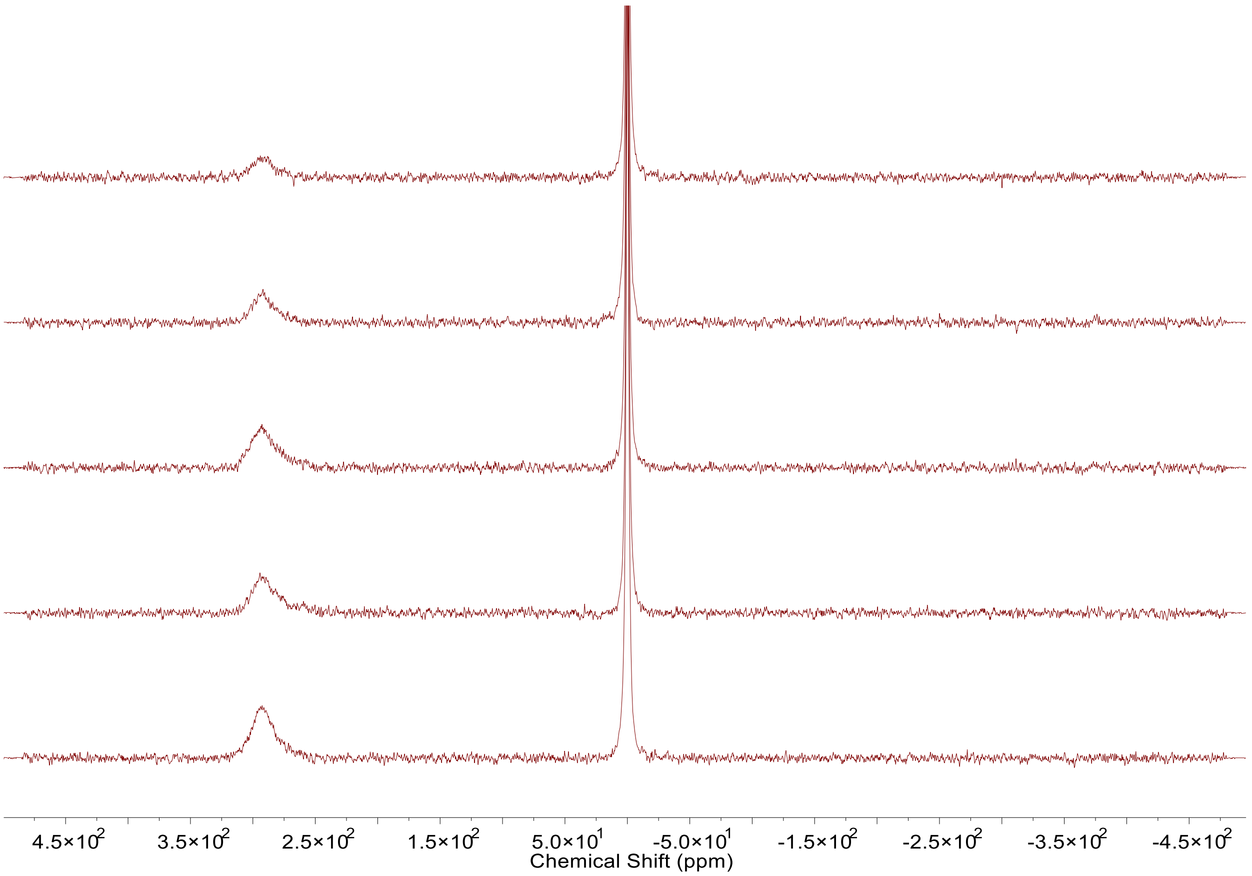

Figure S17. $^{121}Sb\{^1H\}$ NMR spectra (D_2O, 120 MHz, pD 7) for the quantification of the equilibrium constant for the reaction between $K[Sb(OH)_6]$ and potassium gluconate to produce the primary product. Spectra were collected after incubating for 14 d at room temperature. All solutions contained $K[Sb(OH)_6]$ and potassium gluconate in a 1:1 molar ratio. From top to bottom: initial $K[Sb(OH)_6]$ concentration of 15, 25, 35, 45, 55 mM.

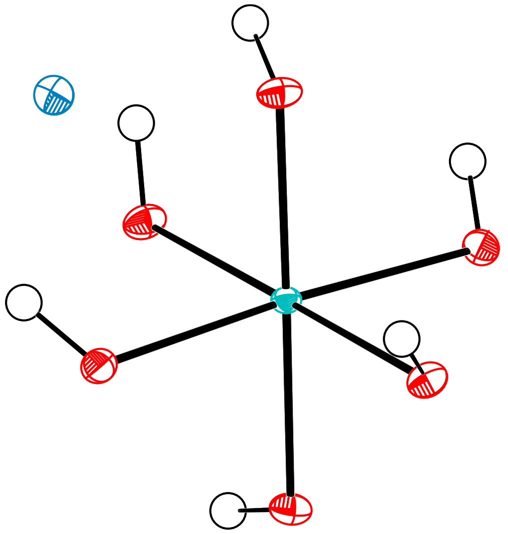

Figure S18. Thermal ellipsoid plot (50% probability) of $K[Sb(OH)_6]$. Color code: Sb teal, O red, K blue, and H grey spheres of arbitrary radius.

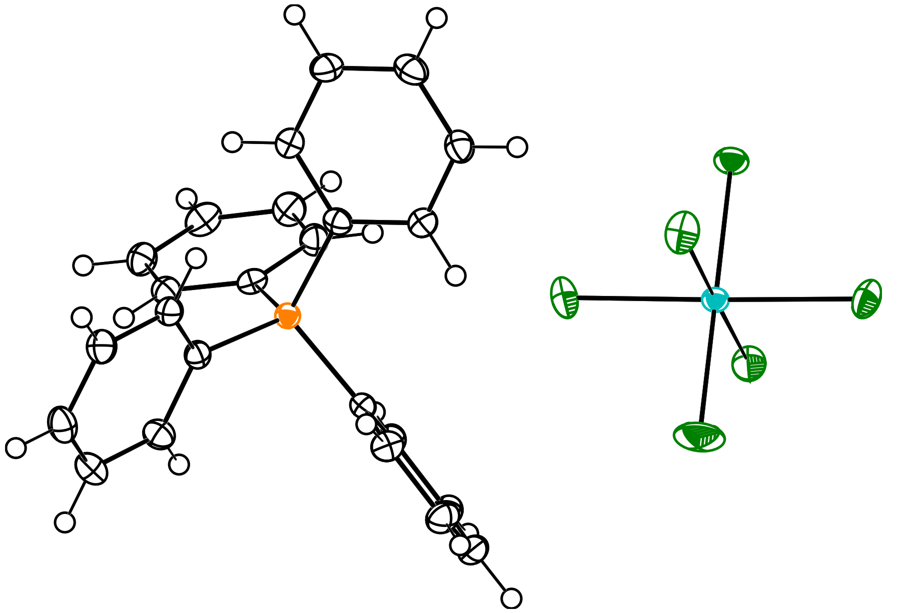

Figure S19. Thermal ellipsoid plot (50% probability) of $(PPh_4)[SbCl_6]$. Color code: Sb teal, Cl green, P orange, C black and H grey spheres of arbitrary radius. The cation resides on a position with site symmetry $\bar{4}$ and the anion resides on a position with site symmetry 4; symmetry-equivalent atoms are depicted to show the full molecules.

Table S1. Crystallographic details for K[Sb(OH)$_6$] and (PPh$_4$)[SbCl$_6$].

Compound	K[Sb(OH)$_6$]	(PPh$_4$)[SbCl$_6$]
Empirical formula	H_6O_6KSb	$C_{24}H_{20}PCl_6Sb$
Formula Weight	262.90	673.82
Temperature (K)	99.97(18)	106(2)
Wavelength (Å)	1.54184	1.54184
Crystal system	Monoclinic	Tetragonal
Space group	$P2_1/c$	$P4/n$
a (Å)	5.72865(13)	12.9370(1)
b (Å)	9.14060(16)	12.9370(1)
c (Å)	10.44891(16)	7.7216(1)
α (°)	90.0045(13)	
β (°)	90.0391(16)	
γ (°)	89.9890(17)	
Volume (Å^3)	547.139(18)	1292.33(3)
Z	4	2
ρ_{calc} (Mg/m^3)	3.192	1.732
Crystal size (mm^3)	0.05 × 0.03 × 0.02	0.19 × 0.13 × 0.08
θ range (°)	4.231 to 68.286	4.834 to 69.845
Total reflections	10738	15557
Unique reflections	1001	1235
Parameters	80	76
Completeness	100	100
R_{int}	0.0375	0.0404
R_1 ($I > 2\sigma$)	0.0117	0.0183
R_1 (all data)	0.0118	0.0184
wR_2 ($I > 2\sigma$)	0.0299	0.0470
wR_2 (all data)	0.0299	0.0471
Goodness of fit, *S*	1.088	1.086

References

[1] F. Frézard, P.S. Martins, M.C.M. Barbosa, A.M.C. Pimenta, W.A. Ferreira, J.E. de Melo, J.B. Mangrum, C. Demicheli, J. Inorg. Biochem. 102 (2008) 656-665.
